# TNL genes in peach: insights into the post-LRR domain

**DOI:** 10.1186/s12864-016-2635-0

**Published:** 2016-04-30

**Authors:** Cyril Van Ghelder, Daniel Esmenjaud

**Affiliations:** INRA, UMR 1355 Institut Sophia Agrobiotech, 06900 Sophia Antipolis, France; University Nice Sophia Antipolis, UMR 7254 Institut Sophia Agrobiotech, 06900 Sophia Antipolis, France; CNRS, UMR 7254 Institut Sophia Agrobiotech, 06900 Sophia Antipolis, France

**Keywords:** TIR-NB-LRR, Peach, Genome, *Prunus*, Resistance, Post-LRR domain, NB-LRR superfamily

## Abstract

**Background:**

Plants develop sustainable defence responses to pathogen attacks through resistance (*R*) genes contributing to effector-triggered immunity (ETI). TIR-NB-LRR genes (TNL genes) constitute a major family of ETI *R* genes in dicots. The putative functions or roles of the TIR, NB and LRR domains of the proteins they encode (TNLs) are well documented, but TNLs also have a poorly characterised C-terminal region, the function of which is unknown in most cases. We characterised this prevalent stress-response protein family in a perennial plant, using the genome of peach (*Prunus persica*), the model *Prunus* species. The first TNL gene from this genus to be cloned, the *Ma* gene, confers complete-spectrum resistance to root-knot nematodes (RKNs) and encodes a protein with a huge C-terminal region with five duplicated post-LRR (PL) domains. This gene was the cornerstone of this study.

**Results:**

We investigated the role of this C-terminal region, by first describing the frequency, distribution and structural characteristics of i) TNL genes and ii) their PL domains in the peach genome, using the v1.0 Sanger sequence together with the v2.0 sequence, which has better genome annotation due to the incorporation of transcriptomic data. We detected 195 predicted TNL genes from the eight peach chromosomes: 85 % of these genes mapped to chromosomes 1, 2, 7 and 8. We reconstructed the putative structure of the predicted exons of all the TNL genes identified, and it was possible to retrieve the PL domains among two thirds of the TNL genes. We used our predicted TNL gene sequences to develop an annotation file for use with the Gbrowse tool in the v2.0 genome. The use of these annotation data made it possible to detect transcribed PL sequences in two *Prunus* species. We then used consensus sequences defined on the basis of 124 PL domains to design specific motifs, and we found that the use of these motifs significantly increased the numbers of PL domains and correlative TNL genes detected in diverse dicot genomes. Based on PL signatures, we showed that TNL genes with multiple PL domains were rare in peach and the other plants screened. The five-PL domain pattern is probably unique to *Ma* and its orthologues within *Prunus* and closely related genera from the Rosaceae and was probably inherited from the common ancestor of these plants in the subfamily Spiraeoideae.

**Conclusions:**

The first physical TNL gene map for *Prunus* species can be used for the further investigation of *R* genes in this genus. The PL signature motifs are a complementary tool for the detection of TNL *R* genes in dicots. The low degree of similarity between PL domains and the neighbouring LRR exons and the specificity of PL signature motifs suggest that PL and LRR domains have different origins, with PL domains being specific to TNL genes, and possibly essential to the functioning of these genes in some cases. Investigations of the role of the oversized *Ma* PL region, in ligand binding or intramolecular interactions for example, may help to enrich our understanding of NB-LRR-mediated plant immunity to RKNs.

**Electronic supplementary material:**

The online version of this article (doi:10.1186/s12864-016-2635-0) contains supplementary material, which is available to authorized users.

## Background

Throughout their lives, plants have to deal with pressures exerted by diverse pathogens, including viruses, bacteria, oomycetes, fungi, and nematodes. Their survival requires the development and maintenance of effective, sustainable defence responses to these biotic stresses. The first line of defence to pathogen attacks involves the early detection of pathogen-associated molecular patterns (PAMPs) through PAMP triggered immunity (PTI) [1]. Pathogens secrete avirulence factors or effectors that manipulate plant immunity and suppress PTI. These factors, also known as *Avr* gene products [2], are then detected directly or indirectly, by the plants, through a second line of defence known as effector-triggered immunity (ETI). ETI involves specific resistance (*R*) genes [1] and genes encoding nucleotide binding–leucine rich repeat (NB-LRR) proteins are the principal class of *R* genes. A wide range of NB-LRR genes have been identified: about 150 in *Arabidopsis* [3], 400 in rice [4] and in poplar [5] and more than 500 in grapevine [6].

NB-LRR genes can be further classified on the basis of their N-terminal domains, into the Toll/interleukin-1 receptor (TIR) NB-LRR and non-TIR NB-LRR (mostly coiled-coil (CC) NB-LRR (CNL)) families [2]. The TIR-NB-LRR family seems to be older than the non-TIR NB-LRR family [7]. TIR-NB-LRR genes (TNL genes) are rare in monocots [8] in comparison with dicots, in which they seem to have emerged earlier in perennials than in annuals [6, 9, 10]. Most of the well-characterized cloned TNL genes [11] belong to *Arabidopsis* [12], but a few originate from plants of agronomic interest, such as potato [13], plum [14] or flax [15]. TNL genes may control plant pathogens as diverse as viruses (*N*/TMV) [16], bacteria (*RPS4*/*Pseudomonas syringae*) [17] and eukaryotes, such as fungi (*L6*/*Melampsora lini*) [15] and nematodes (*Gro1-4*/*Globodera rostochiensis, Ma*/*Meloidogyne* spp.) [13, 14].

TNLs (the proteins encoded by TNL genes) have a conserved organisation into three major domains: the TIR, NB, and LRR domains (in order, in an N-terminal to C-terminal direction). The N-terminal TIR domain, identified by homology with the Drosophila cytoplasmic Toll domain, is involved in downstream protein signalling and pathogen recognition, as shown for the flax *L* gene [18]. TNLs, like the product of the *N* gene in tobacco, form oligomers by direct TIR–TIR interaction [19], as an early event in pathogen detection [20]. The NB domain, a central component of TNLs, is involved in an intramolecular interaction with the TIR and LRR domains and in an extramolecular interaction with ATP/ADP [20]. ATP binding activates TNLs, triggering signalling pathways via conformational changes. Following ATP hydrolysis, the TIR domain activates a downstream signal cascade leading to the plant hypersensitive response (HR). A smaller NLL domain of conserved size, located between the NB and LRR domains, links these two domains through a probable role in TNL protein folding.

The leucine-rich repeat (LRR) domain is located closer to the C-terminus. It is more polymorphic than the N-terminal domains and is subject to diversifying selection [21]. Pathogen effectors are detected by the LRR domain [22–24], and, possibly, by the TIR domain [18]. These effectors may interact directly with the corresponding R gene, as for Avr-Pita from the rice blast fungus, which recognises the Pita R gene of rice [25]. However, the interaction may also be indirect, mediated by plant cofactors or another plant gene, as hypothesized in the ‘guard’ [2], ‘decoy’ [26], ‘bait and switch’ [27, 28] and ‘integrated decoy’ [29] models. A high frequency of genes involved in indirect recognition would account for the limited numbers of R genes relative to the high diversity of pathogens and corresponding effectors. For instance, the *Arabidopsis* RPM1 protein recognizes two avirulent proteins, AvrRpm1 and AvrB, via interaction with the RIN4 protein [21]. Conversely, a single Avr product may interact with several R proteins, as for AvrRps4 and the TNL R proteins RPS4 and RRS1 [30, 31].

The proteins encoded by NB-LRR genes often end in a C-terminal (CT) region comprising other domains of variable structure generally referred as “CT domains”. TNLs have a longer CT region than CNLs [3]. RRS1-R has a large C-terminus including a DNA binding WRKY domain [32, 33] and functions in tandem with RPS4.

In *Prunus,* the TNL gene *Ma* contains a huge CT region with five duplicated exons (each corresponding to a domain in the protein) designated PL (post-LRR) 1 to PL5 [14]. The deduced amino-acid sequence of this PL region of the corresponding protein, the function of which is completely unknown, is longer than the rest of the gene (i.e. TIR, NB, NLL and LRR domains). Moreover, nothing is known about the frequency, distribution and putative structure of this region in its *Prunus* background, represented by the peach (*Prunus persica*) genome (2n = 2x = 16). The *Ma* gene not only has a very unusual structure, it also has a unique biological feature in that it confers complete-spectrum resistance to root-knot nematodes (RKN) of the genus *Meloidogyne* [14].

In peach, two genome versions (v1.0 and v2.0) were obtained from a double-haploid genotype of the cultivar ‘Lovell’. In the v2.0 version, the eight chromosomes cover 225.7 Mb and contain 26,873 protein-coding gene loci [34]. The first objective of our study was to determine the *R* gene repertoire of the *Prunus* genus and the abundance of TNLs with atypical architectures and, in particular, architecture similar to that of the *Ma* gene. To this end, we first identified the complete set of predicted TNL-related sequences in the peach genome, using both the v1.0 [34] and v2.0 genomes, and established the first physical map of these genes for *Prunus* species. The second objective was to study the CT region of TNLs, investigating the frequency, distribution and structural characteristics of PL domains in peach. We took advantage of the *Ma* gene and its unusual PL region, in our analysis of the structure of PL domains in the peach genome and in plant genomes more generally. We identified specific motifs and structural characteristics of PL sequences of use for the further detection and identification of these sequences. Based on our findings, we suggest some possible origins and functions for PL domains.

## Results

### Identification, characterization and distribution of TNLs

**Fig. 1:**
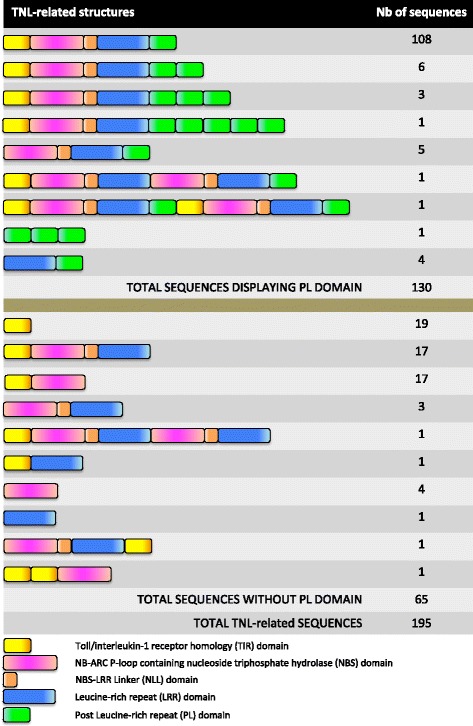
Summary of the structure of the 195 TNL-related predicted genes of the peach genome. The TIR, NB, NLL, LRR and PL domains are shown in yellow, pink, orange, blue and green, respectively. Numbers of TNL-related predicted proteins of each structure are shown in the right column

We carried out BLAST P analysis on the predicted peptides database for the peach genome v1.0, with various relevant sequences as queries, to extract the putative peach TNL proteins. With this screening method, we retrieved 205 predicted TNL proteins from the predicted peptide dataset. The complete structure of all predicted TNLs was characterised and refined by retrieving the associated nucleotide sequences together with a 5 kb 5’ extension and a 5 kb 3’ extension. Analyses of these extended genomic sequences with relevant gene-predicting software suites revealed the presence of unidentified or mispredicted exons, thereby improving v1.0 gene sequence predictions. Fgenesh [35] was the only program to predict the correct intron-exon structure of the *Ma* gene (Additional file 1). The translated DNA coding sequences were then analysed with the InterProScan tool [36], to delineate the various domains in each TNL genes. These procedures made it possible to decrease the total number of TNL genes to 195. This set of TNL genes was also mapped onto the v2.0 genome sequence. The full list of TNL-related genes, with their exon/domain structure, positions and accession numbers (used in both versions of the genome) are indicated in Additional file 2. In parallel, we developed a TNL-specific generic format file (gff3 file, see Additional file 3) to facilitate the comparison between our predictions and the v2.0 genome annotation. We retained the accession numbers from v1.0 of the genome, making it possible to relate the two accession glossaries directly. A comparison of the different types of structures among these TNL genes in the peach genome (Fig. 1) showed that 55 % (108/195) of these genes consisted of five exons corresponding to the following domains assembled in this specific order: TIR, NB, NLL, LRR and PL. The PL domain was present in 67 % (130/195) of the TNL-related sequences. More precisely, 92 % (120/130) of the TNL genes displaying a complete set of classical domains (TIR, NB, NLL and LRR) also had at least one PL domain. Only 12 of the TNLs had repeats of two or more PL domains and only one peptide (ppa21441m), with the highest degree of similarity to the Ma protein, displayed a five-PL domain pattern. Incomplete TNL sequences, often due to recombination processes and frameshift mutations, have also been found and transcriptomic data for the v2.0 sequence have ruled out the possibility of these sequences being pseudogenes. There seems to be a conserved general pattern with a few atypical structures resulting from exon duplication or deletion.

**Fig. 2:**
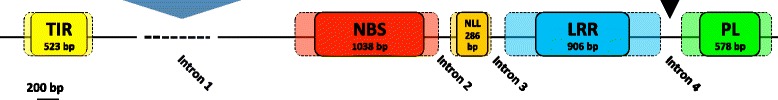
Typical pattern of a TNL in the peach genome. The mean size of each monoexonic domain is indicated. Transparent shapes illustrate the standard deviation of the size of each domain. Size of intron 1 is variable. The black triangle in intron 4 indicates a microsatellite sequence

Based on the complete data for the peach genome, we were able to define a typical TNL pattern (Fig. 2). Each single exon of a TNL gene corresponds to a single domain in the corresponding protein and the mean sizes of the TIR, NB and NLL exons were 523, 1038 and 286 bp, respectively. The exons corresponding to the LRR and PL domains had mean sizes of 906 and 578 bp, respectively. These domains displayed greater size variation than the N-terminal domains, consistent with their greater polymorphism and the difficulties encountered in their accurate prediction. The N, BS4 [GenBank:AAR21295] [37], and RPS4 [GenBank:CAB50708] [38] genes, used as reference TNLs, have a single exon encoding both the LRR and PL domains. This feature was rare in the peach genome, in which the intron between the LRR and PL exons was absent from only 12 % of TNL-PL-related sequences. This intron includes a microsatellite sequence in about 60 % of the TNL-PL-related sequences.

**Fig. 3:**
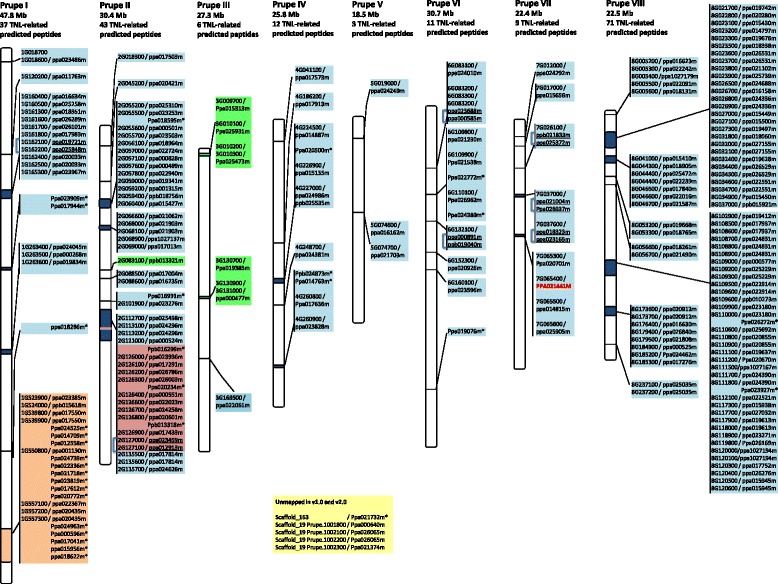
Location of the predicted TNL-related genes in the physical map of the peach genome v2.0. The map position of the 195 total TNL-related proteins is depicted within the eight *Prunus persica* (Prupe) pseudo molecules (chromosomes). Accession numbers are related to the genome glossaries of the v2.0 and v1.0 when available. Merged predicted peptides identified through corrected predictions are underlined and highlighted with brackets. The size of each scaffold (in Mb) and the respective number of TNL-related predicted peptides are shown above each scaffold. The *Ma* orthologue is indicated in red upper case. Unmapped genes in the v1.0 and mapped in the v2.0 are in green boxes. The red box shows re-localisation of TNL genes in the v2.0. Predicted peptides that are not indicated anymore in the v2.0 are labelled with an asterisk. Orange and yellow boxes highlight strand inversion of TNLs and unmapped TNLs, respectively

The TNL genes were very unevenly distributed between the eight peach chromosomes. Most TNL genes (85 %) mapped to four chromosomes (1, 2, 7 and 8), and such genes were particularly abundant on chromosome 8, which harboured more than one third of all the TNL genes (Fig. 3). Analyses of the v2.0 sequence did not predict 31 of the TNL gene sequences identified in the v1.0 genome sequence (Additional files 2 and 3 and Fig. 3), and the use of the former sequence resulted in slight changes in the predicted distribution of the other TNL genes. Indeed, a large cluster of TNL genes originally mapped to chromosome 7 was predicted to be located on chromosome 2 (Additional file 4). Similarly, the use of the v2.0 sequence made it possible to map five TNLs, that had remained unmapped in the v1.0 sequence, to be localised to chromosome 3. In this new distribution of TNL genes, the numbers of TNL genes on chromosomes 3, 4, 5, 6 and 7 are similar, and there are TNL gene hot spots on chromosomes 1, 2 and 8. The number of TNLs is not correlated with chromosome size, as illustrated for chromosome 8, which is one of the smallest chromosomes present. The 12 TNL genes with multiple PL domains were evenly spread between chromosomes 2, 4, 6, 7, and 8. In this context, the *Ma* orthologue (ppa021441m), with its five-PL domain pattern on chromosome 7, appears to be unique within the peach genome.

### Structure of TNL domains

#### TIR, NB, NLL and LRR domains

We extracted each domain individually, to create a set of relevant sequences for use in motif-based analysis. The vast majority of the sequences obtained from the peach genome included sequences encoding TIR and NB domains, and contained five robust motifs each. Most of these motifs have already been described elsewhere, notably for *Arabidopsis* [3] and *Solanum tuberosum* [39]. All the NB domain sequences contained the characteristic P-loop, RNBS-B and GLPL motifs. For the NLL domain, we identified three recurrent motifs present in 95 % of the sequences. As expected, the repeated nature of the sequences of the LRR domain resulted in a higher degree of polymorphism. From the LxxLxL motif, we developed LRR signatures (Table 1) supported by robust e-values and characterised by amino-acid sequences also highly conserved in other plant species.

**Table 1:** TIR, NB, NLL, LRR, and PL motifs detected from TNLs within the peach genome and from reference TNL genes

TNL sequence numbers	Motif	Signature	MEME motif (consensus sequence)	E-value	Sequences displaying the motif	Similar to (Meyers et al.) [3]	Similar to (Jupe et al.) [39]
	TIR1	VFLSFRGxDTRxxFxxHL	YDVFLSFRGEDTRKGFTDHLYHALXXKGI	3.6e-2895	98 %	TIR-1	motif 18
	TIR2	DDxxLxRG	TFRDDDELERG	2.9e-634	95 %		
146	TIR3	YAxSxWCLDEL	YSPELLKAIEESRISIIVFSKNYASSTWCLDELVKIL	3.0e-3730	99 %	TIR-2	motif 15
	TIR4	PxFYxVDPSxVRxQ	VLPIFYDVDPSDVRKQTGSFA	1.2e-2248	99 %	TIR-3	motif 13
	TIR5	KVxxWRxAL	DKEKVLRWRAALTEVANLSGW	2.9e-1463	92 %		
	NB1	GIWGMGGIGKTT	RMVGIWGMGGIGKTTIAKAVYNSISHKFE	8.3e-2337	100 %	P-loop	motif 1
	NB2	GSRIIITT	GKDWFGPGSRIIITTRDKHLL	9.0e-1589	100 %	RNBS-B	motif 5
120	NB3	GLPLAL	YLELSKRVVDYAGGLPLALKVLGSSLYGR	8.1e-1978	100 %	GLPL	motif 3
	NB4	FLDIACF	LDDMEKEIFLDIACFFKGK	2.7e-1129	92 %	RNBS-D	-
	NB5	MHDL	NKLWMHDLLQEMGREIVREE	1.9e-1115	92 %	MHDV	motif 7
	NLL1	FxxMxxLxxL	EAFSKMKNLRLLILSNV	5.8e-1047	95 %	-	-
146	NLL2	LxxLxWxxxPLxxLP	LRWLCWSGYPLKSLP	2.2e-1037	95 %	-	-
	NLL3	FxPxxLxxLxxMxxS	SNFQPEKLVELNMPYSKLRQL	2.6e-1017	95 %	-	-
	LRR1a	PDFxxxPNLxxLxLxxCxxL	TKTPDFSGIPNLERLNLEGCTSLVEVHPSI	2.8e-1638	91 %	-	-
	LRR1b	LxxLxxLxLxGCxxLxxLP	LKSLETLILSGCSKLEKLPEI	1.1e-789	91 %	-	-
118	LRR2a	LxxLxLxGTxIxxLPxSI	MESLKELDLSGTAIRELPSSI	4.0e-767	92 %	-	-
	LRR2b	LxxCxxLxxLPxxI	NLKDCKNLLKSLPSSI	2.4e-461	90 %	-	-
	PL-1	IPxWF	PGSEIPEWFSHQSVGD	1.0e-643	95 %	-	-
124	PL-2	GxAxCxV	KWMGLALCAVFE	3.2e-386	89 %	-	-
	PL-3	VKKCGxxL	VKKCGVRLVYEQDVEELNQT	9.0e-704	83 %	-	-

Numbers of domain sequences used to detect the motifs are indicated in the left column. Each motif is characterised by its specific signature, its MEME consensus sequence, its e-value and the percentage of sequences displaying it. Some motifs from TIR and NB domains correspond to those described in the literature. Motifs are reported in sequence order

#### PL domains

**Fig. 4:**
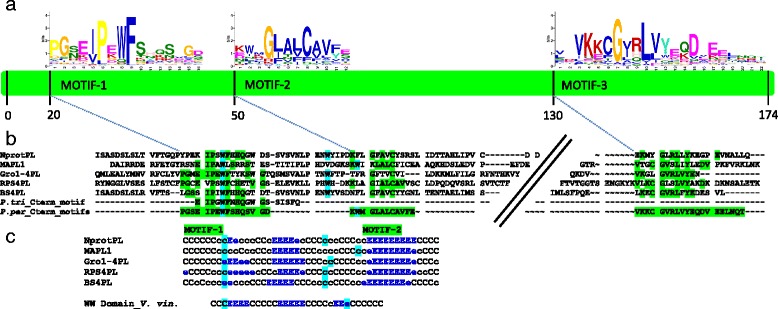
Location and structure of the three conserved motifs defined in the PL domain sequence. **a** The green shape outlines the position of the three motifs with their average start position. **b** Alignment of the five reference PL domains, the motifs described in *Populus trichocarpa* (*P. tri*_Cterm_motif) [5] and the signature found in *P. persica* (*P. per*_Cterm_motifs). Amino-acid identity is highlighted in green and essential tryptophans (W) are identified in blue. **c** HHpred secondary structure prediction of the 5 PL reference domains. C signifies a coil or loop residue. Predicted stands for extended beta sheet (E) are surrounded by the tryptophans (in blue). The putative WW domains (PFAM00397) display the same structural characteristics. The WW domain sequence used (ww Domain_V.vin) is extracted from the perennial species *Vitis vinifera* (XP_010664484.1)

We initially defined the PL domains as sequences located in C-terminus of the TNL-related peptides and not matching any known protein domain. We extracted all the PL domain amino-acid sequences and added the corresponding PL domains from the reference TNLs, N, Ma, BS4, RPS4 and Gro1-4 [GenBank:AAP44390] [13]. By combining expert annotation, alignment and size selection, we obtained a complete set of 124 PL domains that we considered reliable for use in subsequent motif detection. Through MEME analysis, we developed three specific signatures of the PL domain (Table 1), which were recovered in 95, 89, and 83 % of the PL sequences, respectively. When present, these motifs were always detected in the same order from the N- to the C-terminus of the protein. The three motifs included highly conserved amino-acids (Fig. 4a) also present in the PL sequences of the five TNL reference proteins (Fig. 4b). The first motif (PL-1) is located at the N-terminal end and is characterised by a highly conserved “proline-X-tryptophan-phenylalanine” sequence. Its hydrophobic region includes conserved proline and aromatic residues (W, F) and is surrounded by polar, negatively charged amino-acids (D or E) upstream, and by polar, positively charged amino-acids (K, R or H) downstream. The second motif (PL-2), which begins close to the end of the PL-1 motif, contains hydrophobic amino-acids and is characterised by conserved glycine and cysteine residues. Secondary structure predictions indicated the presence of beta-sheet structures, particularly between motifs 1 and 2 and, more precisely, between two tryptophan residues separated by 15–25 amino-acids. This prediction of secondary structure within PL domains is illustrated in Fig. 4c for the reference TNLs. For the five PL repeats encoded by the *Ma* gene, this structure was predicted to occur once for the PL1 and PL2 domains and twice for the PL3, PL4 and PL5 domains (Additional file 5). A sequence length polymorphism between motifs 2 and 3 accounts for the variable size of this domain. Motif 3 contains hydrophobic and polar amino-acids located at the C-terminal end of the domain and the protein.

### TNL gene expression

**Fig. 5:**
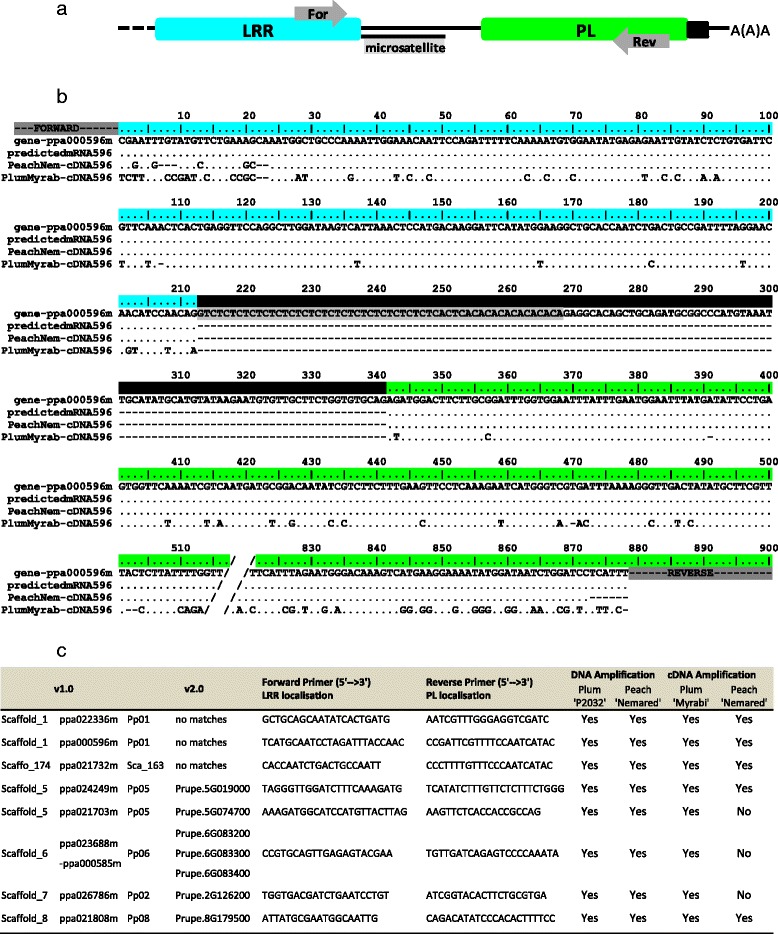
Detection of TNL transcripts using PL sequences. **a** C-terminal representation of TNL sequences with LRR and PL domains in blue and green, respectively. Introns are identified with black lines and primers are positioned with grey arrows. **b** Alignment of the genomic sequence, the mRNA predictions, and the peach and plum cultivar transcripts for C-terminal region of the ppa000596m TNL gene. Dots indicate identical nucleotides and dashes represent non coding sequences. Features follow the same pattern as described in (**a**). **c** List of the transcribed TNL genes in the peach and/or plum cultivars and the associated primers used

We amplified PL sequences from cDNA, by PCR, to assess the expression of TNL genes and their PL domains in *Prunus* spp. We chose 30 candidate TNL genes from throughout the peach genome that met the requirements shown in Fig. 2, focusing particularly on genes with an intron separating the sequences encoding the LRR and PL domains. Using our sequence predictions, we designed, for each candidate, specific primer pairs binding to the “C-terminal” ends of the LRR and PL exons, respectively (Fig. 5a). For representative material from the genus *Prunus*, we used cDNA from the peach cultivar ‘Nemared’ from the subgenus *Amygdalus* and cDNA from the plum cultivar ‘Myrabi’ from another major *Prunus* subgenus (*Prunophora*). We designed primers amplifying specific fragments from both the genomic DNA and cDNA of the two *Prunus* cultivars. We retained eight candidates, distributed over six chromosomes and expressed in peach and/or plum (Fig. 5c). Three of these candidate genes (ppa022336m, ppa000596m and ppa021732) were amplified despite not having been predicted from the peach genome v2.0 sequence. Otherwise the ppa021732m gene could not be mapped to any of the v2.0 chromosomes and the ppa026786m gene was predicted to be located on chromosome 7 on the basis of the v1.0 sequence, but on chromosome 2 on the basis of the v2.0 sequence. A complete PL sequence was expressed for both genes, as demonstrated by the sequencing of PCR products (Additional file 6) (EMBL accession numbers LT555556 to LT555568). For each candidate, the alignment of the peach ‘Nemared’ and plum ‘Myrabi’ cDNA sequences (when available) with their genomic sequences and their sequences as predicted from mRNA revealed the correct location of the intron between the LRR and PL exons (Fig. 5b). As expected, a lower level of polymorphism has been observed between the genome and the ‘Nemared’ peach sequence than between the genome and the ‘Myrabi’ plum sequence (Fig. 5b).

### Phylogenetic analysis

**Fig. 6:**
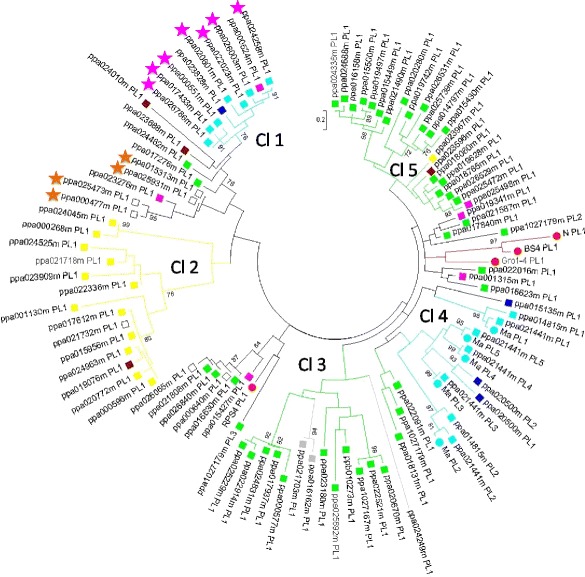
Maximum Likelihood Phylogenetic analysis of the PL sequences from predicted TNL peptides. 94 PL domain sequences (labelled with a square) were used, alongside with nine PL domains from the functional resistance genes N, BS4, RPS4, Gro1-4 and Ma (labelled with a red circle, except Ma which is labelled with a light blue circle). PL domains from TNLs located on scaffolds 1, 2, 4, 5, 6, 7 and 8, in the v1.0 assembly, are labelled in yellow, purple, dark blue, grey, brown, light blue and green respectively. Unmapped sequences are labelled in white. Predicted peptides marked with purple and orange stars are attributed to chromosomes 2 and 3 respectively in the v2.0. The protein labels contain the genome predicted peptide identifier followed by the PL domain number. Bootstraps over 70 (out of 100) are shown

A phylogenetic analysis of the PL domain, conducted with 103 trimmed sequences, showed that PL sequences could be organised into five clades (Fig. 6). As expected from the TNL gene distribution in peach (Fig. 3), most PL sequences mapped to chromosomes 1, 2 and 8, and these sequences could be grouped into identifiable clades. Clade 2 groups together PL sequences from chromosome 1. Clades 1 and 4 were both initially located in chromosome 7 on the basis of the v1.0 genome sequence. Analysis of the v2.0 sequence relocated clade 1 to chromosome 2, whereas the sequences of clade 4, including the *Ma* gene orthologue in particular, mapped to chromosome 7. The PL sequences from chromosome 8 (which had the highest proportion of PL sequences) were split between clades 3 and 5. These data highlight the correlation between the similarity and physical location of the PL domain sequences. This correlation made it possible to attribute the unmapped TNLs ppa026065m and ppa000640m from clade 3 to chromosome 8 and the unmapped TNL ppa021732m from clade 2 to chromosome 1. Similarly, the four unmapped TNLs from the same subclade unmapped in the v1.0 sequence, ppa015313m, ppa025931m, ppa025473m and ppa000477m, were localised to the same cluster on chromosome 3 of the v2.0 sequence (Additional file 4).

Within the *Ma* gene cluster, the higher degree of conservation of the PL sequences between *Ma* [GenBank:CAR94514] and its peach orthologue (ppa021441m) than between the *Ma* peach orthologue (ppa021441m) and its peach paralogue (ppa014815m), all belonging to clade 4, indicates that the separation of the peach and plum lineages occurred after the TNL duplication. As expected, the three reference TNL genes from the Solanaceae family (*BS4*, *N* and *Gro1-4*) were grouped together into the same subclade, whereas the crucifer *RPS4* TNL gene belonged to another subclade. Nevertheless, these outer groups are not isolated from the peach TNL genes.

### PL detection among genomes

MAST and BLASTP analyses were conducted with the PL motifs and the Plant Genome Database, to determine the number of TNL-related protein genes that could be detected in ten reference genomes of various sizes. The *P. persica* dataset was used to assess the relevance of our approach, Using PL motifs, we were able to detect 72 % (94 of 130) of all peach TNLs with one or several PL domains (Table 2). Indeed, the automated prediction for v1.0 did not detect PL domains for any of the 36 missing sequences that were later annotated as PL-carrying TNL genes. Nevertheless, a combination of the use of transcriptomic data and the reannotation of the v2.0 genome sequence resolved most of the mispredicted PL domains. In dicots genomes, between 50 and 87 % (Table 2) of TNL sequences could be detected using PL signatures. For example, in the *P. trichocarpa* genome [5] 69 of the 91 TNL related-genes identified presented PL signatures. Thus, at least three quarters of the TNLs of this species carry a PL domain. The proportion of TNL genes with a PL domain was highest in the potato genome (87 %). By contrast, no PL domain sequences were detected in the three Poaceae species (*O. sativa, S. bicolor* and *Z. mays*), consistent with the almost total absence of TNLs in monocots. Moreover, no non-TNL sequences were found to have PL motifs with a consistent e-value (<0.01), suggesting that these motifs are specific to TNLs. The detailed results of the BLASTP analysis are reported in Additional file 7. Furthermore, the use of our motifs led to the detection of particular PL domain repeats in peptide databases. We detected a few proteins with atypical structures in *V. vinifera* [Grape Genome Browser:GSVIVT01037222001] and in *P. trichocarpa* [Plant GDB:POPTR_0019s09760.1], with LRR-PL-LRR-PL-LRR-PL and TIR-NBS-LRR-PL-PL domain chains, respectively (Additional file 7). Surprisingly, the structure of the *Prunus* Ma protein, with its five-PL domains, remained unique, as no other predicted TNL proteins with a similar pattern of multiple PL domains were identified in any of the other plant genomes tested. An orthoMCL analysis on 31 plant genomes conducted with the protein orthologous to Ma in peach (ppa021441m) revealed an orthoMCL group (GRP18645) including the other three members of the ppa021441m cluster (ppa014815m, ppa020701m, ppa025905m) (Fig. 3) and six other members from the apple protein dataset (*Malus domestica*) [GDR:MDP0000267764, MDP0000176003, MDP0000272063, MDP0000158765, MDP0000264373, MDP0000143700]. MDP0000176003 is presumably the Ma apple orthologue (lacking the TIR domain), as it has a similar five-PL domain structure. Finally, we tried to detect these motifs in other *R* genes encoding proteins with LRR domains, such as transmembrane-LRR and CNL proteins. We detected no PL motifs in any of the genes tested (complete list of genes in Additional file 8).

**Table 2:** Number of TNL-related proteins recovered in seven dicots and three monocots genomes

	Plant species	Genome size	Nb NBS-LRR	Nb TNL	Source	Nb TNLs detected using the PL motifs	% of TNL-related protein containing PL signatures
Dicots	*Prunus persica*	265	437	185	[34]	94	51
	*Populus trichocarpa*	485	402	91	[5]	69	76
	*Vitis vinifera*	487	233	97	[6]	62	64
	*Arabidopsis thaliana*	135	149	94	[3]	67	71
	*Solanum tuberosum*	844	438	77	[39]	67	87
	*Glycine max*	1115	319	116	[58]	50	43
	*Medicago truncatula*	500	333	156	[59]	78	50
Monocots	*Oryza sativa indica*	466	653	0	[60]	0	0
	*Sorghum bicolor*	730	274	0	[61]	0	0
	*Zea mays*	2400	109	0	[62]	0	0

Numbers of TNLs harbouring PL motifs in the Plant GDB and their ratio are indicated from total putative TNLs reported in the literature

## Discussion

### Distribution and characterization of TNLs

Following on from the 2003 study by Meyers et al. in *Arabidopsis* [3], many studies have made use of the increasing number of plant genomes available to describe the distribution and diversity of NB-LRR genes in genomes [5, 39–41]. We focused on TNL-related sequences as the major group within the NB-LRR superfamily. We first identified the predicted TNLs within the peach genome, which is considered to be representative of the genus *Prunus*. We found that the 195 predicted TNL sequences retrieved from the peach genome were very unevenly distributed between the eight chromosomes, with 85 % located on four chromosomes (1, 2, 7 and 8), one of which (chromosome 8) carried more than a third of all the predicted TNL sequences. As expected, a large proportion (86 %) of these TNLs were grouped into clusters of at least two duplicated sequences separated by short physical distances and displaying a high degree of sequence similarity. Three of these clusters included at least 20 TNLs. These data thus confirm the involvement of tandem or segmental gene duplication and recombination as major mechanisms of *R* gene expansion in peach and other plant species [42]. In perennial plants, which have a much longer generation time than annuals, and particularly in those of the Rosaceae, these mechanisms may constitute an adapted response to pathogens, contributing substantially to the great diversity of this group of proteins, as observed in apple [40]. We used the large set of sequences obtained from peach to identify motifs specific to the various TNL domains. Our findings confirm the conserved motifs previously identified for the TIR and NB domains by Meyers et al. [3] and Jupe et al. [39] and identify original specific motifs for NLL and LRR sequences.

By contrast, little is known about the C-terminus of TNLs, other than for the WRKY domain [32, 43]. This study, in which peach was used as the reference genome, increases what is known about the frequency, distribution and structural characteristics of PL domains in TNL genes. With this objective in mind, given that automatic PL domain predictions are currently not very reliable, we first re-annotated the complete set of peach TNL sequences. This re-annotation increased the number of PL domain sequences detected within TNL genes from 94 initial sequences (in the v1.0 genome annotation) to 130 final sequences. The v2.0 genome, with the incorporation of transcriptomic data, thus represents a real improvement. Based on these high-quality PL sequences, we were able to identify three conserved motifs flanking polymorphic regions. Using these conserved motifs, we were able to retrieve PL sequences from the vast majority of sequences encoding TNLs with complete classical domains (TIR, NB, NLL and LRR). Beyond *in silico* prediction, we were able to amplify TNL transcripts generated from genes located on different chromosomes. Our results illustrate that (i) TNL transcripts can be identified through the detection of PL domain sequences, (ii) our predictions are relevant even for transcripts from the v2.0 genome sequence, and (iii) the peach genome can be used as a reliable matrix for gene studies in the various species (peach, almond, plum, etc.) of the genus *Prunus*.

The cloning and sequencing of the *Ma* gene from *Prunus* revealed the presence of duplicated PL domains [14], providing us with an opportunity to enhance our knowledge of this particular region. Using peach PL sequences and PL sequences from reference TNL genes, we then carried out the first phylogenetic analysis of the PL domains in the peach genome. We found that the PLs present on the same chromosome evolved with their mother TNL genes, through duplication processes [42], resulting in similar sequences and physical positions. Moreover, TNLs with multiple PL domains were found to be rare, with only 12 TNL genes found to encode proteins with more than one PL domain. The Ma orthologue was the only protein encoded by the peach genome that was found to include five PL domains. The *Ma* gene, as the only functional TNL gene with multiple domains identified to date, remains a reference sequence for structural analyses of PL domains. The phylogenetic localisation of PL domain sequences from the *Ma* cluster and from genes orthologous to *Ma* demonstrated that TNL diversification occurred before the splitting of the *P. persica* lineage from the plum species acting as the *Ma* donor, *P. cerasifera.* Moreover phylogenetic and orthoMCL analyses reveal that the 5-PL *Ma* structure, presumably present in *Ma* orthologous genes from other *Prunus* species, is also present in its apple (*Malus domestica*) orthologue. The genera *Prunus* and *Malus* belong to the Rosaceae family, which can be subdivided into two prevalent subfamilies, Spiraeoideae *(Prunus/Malus*) and Rosoideae (*Fragaria/Rosa*) [44]. Thus, within the Spiraeoideae, ancestor genes with the multiple-PL domain structure of *Ma* predate the separation of the genera *Malus* and *Prunus* [45]. Finally, our results show that exon duplications have led to the multiple-PL domain structure of *Ma* and generated a relatively high level of polymorphism. This polymorphism may have given rise to the putative original function of the duplicated PL region of *Ma*. We found that 60 % of PL-carrying TNLs had a microsatellite sequence in the intron separating the LRR and PL domains, providing clues to a possible modular mechanism of evolution. This polymorphic site is suitable as a molecular marker for the detection and monitoring of *R* genes, for plant breeding purposes, for example.

Our findings confirm that PL domains are basic components of TNLs not only in peach, but also in other dicots. As expected, no PL sequences were detected in the Poaceae species, consistent with the almost total absence of TNLs in monocots [8]. Using the specific PL motifs defined, we were able to recover PL domains with similar frequencies from the TNLs of dicots from various botanical families, and with different genome sizes and NB-LRR pools. In addition to detecting TNLs, this approach also made it possible to determine the prevalence of this domain within dicot species. Indeed, the proportion of PL-carrying TNLs ranged from 43 % in soybean to 87 % in potato (including the functional *R* gene *Gro1-4*) [13]. We can assume that these values are underestimates, due to the difficulties in the annotation of this C-terminal domain, which are likely to be resolved by transcriptomic data. Our results highlight the unusual nature of the PL domain, with its specific signature motifs, providing strong support for the use of this domain as a specific marker of TNLs.

### Putative origin of the PL domain

The location of the PL domain downstream from the LRR domain, as illustrated in peach, suggests that it may be considered a simple extension of the LRR domain. This hypothesis is supported by the finding that the LRR and PL domains are sometimes encoded by a single exon, as for the reference genes BS4, RPS4 and N. This situation occurred at low frequency (12 %) in the peach genome, suggesting that the two domains may originally have been separated, with fusion into a single exon resulting from intron deletion. It is commonly thought that the PL domain originated from an expansion, by (partial) duplication, of the LRR domain. There are two implications of this hypothesis. Firstly, PL domains should also be present in other *R* gene families containing LRR domains, such as transmembrane–LRR (*Cf* genes) [46], kinase-LRR (*Xa21* gene) [47], and CNL (*Mi1-2* gene) [48] genes. However, this was not found to be the case, as none of the genes from these *R* gene families were found to carry PL domains. The second implication is that LRR motifs should also be detected, even residually, in the PL domains and vice versa, whereas this was not the case. As already reported for *Arabidopsis* [3], the C-terminal domain of TNL proteins has no conserved LRR motif. It therefore seems highly unlikely that the PL domain originated from the LRR domain and it seems more probable that the only relationship between these domains is their location within the protein.

### Putative role of the PL domain in ligand binding and/or intramolecular interactions

The absence of PL domains from proteins other than TNLs and their systematic location in the C-terminal position after the LRR domain in TNLs suggest a possible role of these domains in recognition via ligand binding. Recent studies on the CNL gene RGA5 have highlighted the importance of a C-terminal domain located after the LRR domain. Indeed, the heavy metal interaction domain concerned is directly involved in the binding and recognition of rice blast fungus effectors [49]. The PL domain is not related to the C-terminal sequence of RGA5, but its specific amino-acid sequence or structural features may be consistent with a particular role or function. Interestingly, all three PL-specific motifs are present together in the functional TNL genes Ma (several PL repeats), Gro 1–4, RPS4, BS4 and N (single PL domains). These three conserved motifs, two of which (motifs 1 and 2) were identified by Dodds et al. [23] and confirmed by Claverie et al. [14], are separated by a conserved beta-fold structure. The WW domain has several features in common with the PL domain in that it consists of two tryptophan residues separated by 20–23 amino-acids, with one conserved proline and three beta-sheet structures. WW domains are involved in binding to particular proline rich-motifs [AP]-P-P-[AP]-Y in other proteins [50]. Such similarities, together with the characteristics of the PL domain, suggest that the PL domain may be involved in ligand recognition. The PL domain may also be involved in the intramolecular interactions of TNLs, and, in particular, in interactions or joint action with the TIR domain. Like the TIR domain, which is involved in both downstream signalling and ligand recognition [51], PL domains may modulate activity and interact with the TIR domain. Indeed, in the closed three-dimensional structure of TNLs, these two domains are located in close proximity.

TNLs involved in atypical processes, with other specific signatures, such as a signal peptide (L6 from flax) or a WRKY domain (RRS1 from *Arabidopsis*), have no PL domains. This is consistent with a putative general function of PL domains in TNLs located in the cytoplasm. Recent studies on RRS1 [52, 53] have highlighted a particular process involving dimerisation between RPS4 and RRS1 via the TIR domain, but did not attribute a function to the PL domain of RPS4 (named CTD). The possibility of a role for the PL domain in downstream signalling cannot, therefore, be excluded. Indeed in paired R proteins, this domain is present in each of the TNL partners, RPS4 and RPS4B, of the WRKY-carrying proteins, RRS1 and RRS1-B, respectively [30]. However, no RRS1 orthologue and no other TNL with a WRKY domain has been found in the peach genome, suggesting that this particular process may not occur in peach.

Given the small number of sequences with multiple PL domains detected, TNL genes with multiple PL domains should be considered exceptional in plant genomes. The unique five-PL domain structure of *Ma* may be involved in the recognition of effectors or guard proteins and/or in modulation of the plant hypersensitive response. The *Ma* gene confers high, complete-spectrum resistance to all *Meloidogyne* species tested, including the predominant species from the polyphagous root-knot nematode (RKN) complex, *M. arenaria, M. incognita,* and *M. javanica*, and the invasive species *M. enterolobii* [14]. By contrast, other RKN *R* genes from *Prunus*, such as the peach *RMia* gene (acting against *M. incognita* and *M. arenaria*) and the almond gene *RMja* (acting against *M. javanica*), display a more restricted resistance pattern [54, 55]. The complete resistance spectrum of *Ma* may be linked to a broad-spectrum recognition of effectors or guard proteins. We cannot, therefore, exclude the hypothesis that this recognition is mediated by both the LRR domain and one or several repeats in the PL region. The rare event of PL duplication, initiated from PL1, may have generated the steric conformation of the *Ma* TNL, ultimately leading to more sustainable and extensive resistance. In *Prunus*, gene editing with C-terminally truncated or mutated regions should shed light on the role of these PL repeats. Just as the Rx response to the potato virus X coat protein was shown to depend on the C-terminal region of the LRR domain [56], modifications of the PL region may influence the degree or spectrum of resistance conferred by the *Ma* gene.

## Conclusions

The peach genome contains numbers of TNL genes similar to those in other perennial plants with genomes of similar size. The distribution of the genes of this family and of the variants of its specific domains between peach chromosomes confirmed the hypothetical mechanisms of TNL expansion. PL domains were recovered from about two thirds of the TNLs identified. The PL dataset for the peach genome was used to define specific motifs that will be useful complementary tools for detecting and localising TNLs and their variants in plant genomes. PL domains may be required for the function of some TNL proteins, particularly through specific motifs potentially involved in the recognition of effectors or guard proteins. TNL mapping and the definition of PL domain signature sequences should make it possible to identify functional TNLs from peach or, more widely, in other perennial plants and members of the Rosaceae. TNLs with multiple PL domains are rare in peach and, presumably, in other plants and the five-PL domain structure of *Ma* appears to be unique in peach and, more generally, among perennial and annual plants. Investigations of the putative role or function of the oversized PL region of the *Ma* gene in the resistance properties (high, broad-spectrum resistance) should increase our understanding of the mechanisms of resistance involving NB-LRR-based plant immune receptors.

## Methods

### Detection and mapping of the TNL genes

TNLs in the *Prunus persica* v1.0 genome were detected by Blast P analysis with the NCBI BLAST tool available from the GDR website (https://www.rosaceae.org/tools/ncbi_blast). The blast analyses were carried out with the default parameters, except for the description and alignment parameters, which were increased to 500 to visualize as many results as possible in the graphical overview. We used the sequences of the TMV resistance protein N (Q40392.1) [57], the RKN resistance protein Ma (CAR94514.1) [14], the TIR-1, −2, −3, and −4 motifs developed by Meyers et al. [3] and the TIR motifs 13, 15, and 18 developed by Jupe et al. [39], as queries, and the peach genome v1.0 predicted peptides as the database. The N and Ma sequences were chosen because they appeared to be the most relevant for TNL detection. Indeed the *N* gene is the reference TNL gene for plants in general, and the *Ma* gene is the only *Prunus* TNL *R* gene to have been functionally validated. The TIR domain is characteristic of the TNL family. We therefore retained the TIR motifs developed from genome-scale data for *Arabidopsis* and potato. As a complementary tool for the detection of TNL proteins, we used the PL motifs defined in this study to query the database. We retained the predicted peptides with an e-value below 1e-04 for detection with N and Ma, below 0.068 for detection with the TIR motifs and below eight for detection with the PL motifs. The protein sequences retrieved were subjected to InterProScan analysis to confirm that they corresponded to TNLs. We also cross-referenced the data with the complete list of proteins from the predicted proteins database available from the GDR website (ftp://ftp.bioinfo.wsu.edu/species/Prunus_persica/Prunus_persicagenome.v1.0/homology/Prunus_persica_v1.0_vs_swissprot.xls). Finally, all the predicted TNLs recovered were visualised individually with the GDR Gbrowse tool (http://www.rosaceae.org/gb/gbrowse/prunus_persica/). The link between the v1.0 and v2.0 genomes was obtained with the Blast tool. The mRNA sequences predicted from the v1.0 genome were blasted against the v2.0 genome pseudomolecules and scaffolds, to determine intron/exon positions, and ORFs were defined for each exon of each candidate for annotation purposes (Additional file 3). This file must be uploaded onto a local Gbrowse tool, such as IGV, or obtained via the Gbrowse tool of the GDR website (https://www.rosaceae.org/gb/gbrowse/prunus_persica_v2.0.a1/), together with the *Prunus persica* v2.0 genome.

### Identification and structure of TNL-related proteins

The nucleotide sequences of predicted TNL genes were extracted with 5 kb of upstream sequence and 5 kb of downstream sequence, with the GDR Sequence Retrieval tool available from http://www.rosaceae.org/retrieve/sequences. Fgenesh, an HMM-based *ab initio* gene predictor (gene-finding parameters: *Solanum lycopersicum, Vitis vinifera, Hevea brasiliensis*, and *Populus trichocarpa*), was used to predict TNL gene structures. The individual analysis and expert annotation of these predicted genes made it possible to assemble their intron-exon structures. The predicted amino-acid sequences were then analysed with InterProScan 5, which was used to identify the various domains with default parameters and with the whole set of available databases. This approach made it possible to associate exon structures with families of domains.

### PL transcript sequences

Two members of the genus *Prunus*, the peach (*P. persica)* cultivar ‘Nemared’ and the Myrobalan plum (*P. cerasifera*) cultivar ‘Myrabi’ (P.2032), were grown in a growth chamber. Young leaves from each cultivar were collected so as to obtain samples of 100 mg of biological material. Half of each sample was used for DNA extraction [55], the rest being used for total RNA extraction with the TQ RNA Cells Tissues Kit (Talent). An additional DNAse digestion was carried out with Turbo DNA-free (Ambion), and the quality of the final RNA preparation was checked by electrophoresis in a 1.5 % agarose gel. First-strand cDNA synthesis was performed with oligo(dT) primers and the Maxima H Minus first-strand cDNA synthesis kit (Thermo Fisher). PCR was carried out with the MyTaq polymerase kit (Bioline), specific primer pairs (Fig. 5c) and the following steps: initial denaturation for two minutes at 94 °C, followed by 35 cycles of 30 seconds at 94 °C, 30 s at the annealing temperature and one minute at 72 °C, and a final extension step of three minutes at 72 °C. PCR products were run on 1.5 % agarose gels and the fragments observed were extracted from the gel with the Minelute gel extraction kit (Qiagen). Purified PCR products were sequenced with Sanger technology. All experiments were conducted according to the kit manufacturer’s instructions, unless otherwise specified.

### Sequence alignment and generation of a phylogenetic tree for PL domains

We trimmed the 124 sequences to obtain a final alignment composed of 103 PL domains (including 9 PL domains from N (1), Ma (5), RPS4 (1), BS4 (1) and Gro1-4 (1)). The full sequence alignment (246 positions, gaps included) included the three PL motifs. Domain sequences from the five cloned functional reference TNLs, RPS4 [GenBank:CAB50708], N [Swiss-Prot:Q40392], BS4 [GenBank:AAR21295], Gro1-4 [GenBank:AAP44390], and Ma [GenBank:CAR94514], were extracted as previously described. The amino-acid sequences of the domains were manually curated and aligned, using MEGA6 and Muscle with default settings. The phylogenetic tree for PL domains was constructed with MEGA6, using a Maximum Likelihood method with 100 Bootstrap replications and the Jones-Taylor-Thornton model.

### Definition and analysis of specific PL–domain signatures

Using the complete dataset for TNLs, we confirmed the position of each domain by InterProScan analysis, and we extracted each domain individually. For each of the TIR, NB, NLL, and LRR domains, we removed those (i) encoded by more than two exons and (ii) with domain lengths of less than 450, 950, 260, and 750 bp, respectively. We then performed a rough alignment for each domain, to remove potential aberrant sequences. The final set of sequences obtained was analysed with MEME software. As for other TNL domains, we considered a single exon to correspond to a single domain and we arbitrarily decided to consider exons consisting of more than 400 nucleotides as valid PL domain exons. After this initial selection on a size basis, a rough alignment was used to exclude aberrant sequences. MEME software was used to identify specific signatures from sequence alignments. The settings used were as follows: 0 or 1 occurrence of the motif per sequence, with a maximum of five different motifs and a minimum-maximum motif width of 5–30 amino-acids. MAST and BLASTP analyses conducted with default parameters identified PL domains in predicted peptides. BLASTP analyses with the Plant Genome DataBase website (http://www.plantgdb.org/cgi-bin/blast/PlantGDBblast) led to the identification of PL domain-related sequences and, therefore, TNL sequences. The genomes used for this study were those of perennial (peach, poplar, grape) and annual (barrel clover, potato) dicots, monocots (rice, sorghum), a model plant (*Arabidopsis*) and a monocot (maize) and a dicot (soybean) with large genomes. We analysed the secondary structure of the PL domains with HHpred and the Chou Fasman Secondary Structure Prediction Server available on the Expasy resource portal, for the 124 sequences subjected to MEME analysis and the associated consensus sequence.

### Ethics approval and consent to participate

Not applicable.

### Consent for publication

Not applicable.

### Availability of data and material

The datasets supporting the conclusions of this article are either available in the DRYAD repository, doi:10.5061/dryad.dp20h, http://dx.doi.org/10.5061/dryad.dp20h, or in https://www.ebi.ac.uk/ena, http://www.ebi.ac.uk/ena/data/view/LT555556-LT555568 for biological sequences [EMBL accession numbers LT555556 to LT555568] or included within the article and its additional files.

## Additional files

Additional file 1: Structure of the *Prunus* TNL gene *Ma* predicted with the predictors Fgenesh and GENESCAN using various specific gene-finding parameters. The first row shows the exact size of each exon (and associated domain in bp) of the *Ma* gene. The other rows show the exon predictions. NA not appropriate. (DOCX 48 kb) Additional file 2: Exon and domain structures of all predicted TNL-related peptides. Exon sizes are shown above the colour shapes reflecting the different domains. (XLSX 115 kb) Additional file 3: TNL prediction mapped in the *Prunus persica* genome v2.0. This Generic Feature Format Version 3 (GFF3) can be uploaded as a custom track directly in a local Genome browser or the online GDR Gbrowse, together with the genome v2.0. (GFF3 88 kb) Additional file 4: Physical map of the TNL-related genes in the peach genome v1.0 and revision observed in v2.0. The map position of the 195 total TNL-related proteins is depicted within the eight scaffolds (chromosomes). Accession numbers are related to the genome glossary of the v1.0. Merged predicted peptides identified through corrected predictions are underlined and highlighted with brackets. The size of each scaffold (in Mb) and the respective number of TNL-related predicted peptides are shown under each scaffold. The *Ma* orthologue is indicated in red upper case. Unmapped genes in the v1.0 and mapped in the v2.0 are in green boxes. The red box shows re-localisation of TNL genes in the v2.0. Predicted peptides that are not indicated in the v2.0 are labelled with an asterisk. (PDF 167 kb) Additional file 5: Secondary-structure prediction of the PL motif-1. The table shows the consensus sequence obtained from the alignment of the peach PL sequences, the 5 PLs of the Ma protein and the WW-domain consensus sequence obtained from *Vitis vinifera* sequences. H signifies a helix residue. Predicted stands for extended beta sheet (E) are surrounded by the tryptophans (in blue). (DOCX 17 kb) Additional file :6 Transcribed PL sequences. Sequences of the transcribed genes ppa022336m, ppa000596m, ppa021732m, ppa024249m, ppa021703m, ppa023688m-ppa000585m, ppa026786m and ppa021808m for the peach ‘Nemared’ and plum ‘Myrabi’ (= ‘P.2032’) cultivars. (FAS 7 kb) Additional file 7: TNL-related peptides obtained with BLASTp analysis using MEME motifs of the post-LRR domain in the Plant Genome Database website. Results are shown for *Populus trichocarpa, Vitis vinifera, Medicago truncatula, Arabidopsis thaliana, Solanum tuberosum, Glycine max, Oryza sativa, Prunus persica, Sorghum bicolor,* and *Zea mays*. Identified TNL-related sequences, unrelated TNL sequences and unknown sequences are highlighted in green, blue and yellow, respectively. (DOC 138 kb) Additional file 8: Main functional resistance genes described in the literature. The table includes names, host plants, pathogens controlled and structural characteristics of the resistance genes. (XLSX 15 kb)

## Abbreviations

CC: coiled-coil
ETI: effector-triggered immunity
GDR: genome database for rosaceae
NB-LRR: nucleotide-binding site leucine-rich repeat
PAMP: pathogen-associated molecular pattern
PGDB: plant genome database
PL: post-leucine-rich repeat
PTI: pamp-triggered immunity
TIR: toll/Interleukin1 receptor
TMV: tobacco mosaic virus
