# Supplementary material for: TNL genes in peach: insights into the post-LRR domain
Source: BMC Genomics. 2016 Apr 30;17:317. doi: 10.1186/s12864-016-2635-0 (PMC4851768; doi:10.1186/s12864-016-2635-0)
Supplement: Additional file 1: — Structure of the Prunus TNL gene Ma predicted with the predictors Fgenesh and GENESCAN using various specific gene-finding parameters. The first row shows the exact size of each exon (and associated domain in bp) of the Ma gene. The other rows show the exon predictions. NA not appropriate. (DOCX 48 kb) [file 12864_2016_2635_MOESM1_ESM.docx]

| **Predictor / matrix** | **TIR** | **NBS** | **NLL** | **LRR** | **PL1** | **PL2** | **PL3** | **PL4** | **PL5** |
| --- | --- | --- | --- | --- | --- | --- | --- | --- | --- |
| *Ma* exon structure | 519 | 1107 | 279 | 972 | 678 | 789 | 639 | 624 | 537 |
| Fgenesh / *Solanum lycopersicum* | 519 | 1107 | 279 | 972 | 678 | 789 | 639 | 624 | 537 |
| Fgenesh / *Vitis vinifera* | 519 | 1107 | 279 | 795 147 | 678 | 789 | 639 | 624 | 537 |
| Fgenesh / *Nicotiana tabacum* | 519 | 1107 | 279 | 795 147 | 678 | 789 | 639 | 624 | 537 |
| Fgenesh / hevea | 519 | 1107 | 279 | 795 | 678 | 789 | 639 | 624 | 537 |
| Genescan / *Arabidopsis thaliana* | 521 | 1108 | 279 | 795 | 147 121 269 156 | 789 | 639 | 624 | 537 |
| Genescan / *Zea mays* | NA | NA | NA | NA | NA | NA | NA | NA | NA |

**Additional file 1 Structure of the *Prunus* TNL gene *Ma* predicted with the predictors Fgenesh and Genescan using various specific gene-finding parameters.** The first row shows the exact size of each exon (and associated domain in bp) of the *Ma* gene. The other rows show the exon predictions. NA not appropriate.
