# Supplementary material for: TNL genes in peach: insights into the post-LRR domain
Source: BMC Genomics. 2016 Apr 30;17:317. doi: 10.1186/s12864-016-2635-0 (PMC4851768; doi:10.1186/s12864-016-2635-0)
Supplement: Additional file 5: — Secondary-structure prediction of the PL motif-1. The table shows the consensus sequence obtained from the alignment of the peach PL sequences, the 5 PLs of the Ma protein and the WW-domain consensus sequence obtained from Vitis vinifera sequences. H signifies a helix residue. Predicted stands for extended beta sheet (E) are surrounded by the tryptophans (in blue). (DOCX 17 kb) [file 12864_2016_2635_MOESM5_ESM.docx]

| **Peach consensus sequence of the PL MOTIF-1 & 2** | PEWFSHQVGSSVSFELPPSWXGLAL  ----------EEEEE-------HEE |
| --- | --- |
| **Ma gene:**  PL1  PL2  PL3  PL4  PL5 | PAWLSRRSTESTITIPLPHDVDGKSKWIKLAL  HHHH-------EEEE----------HEHHHHH  PWFSEQSSTSSCTVNLRLPPNLHNNEKWAGLS  -----------EEEEEE--------------E  VEWFGHQSSGPSVKIPLPSNLCEDTNWIGLAL  -----------EEEEE-----------EEEEE  FKWLYRMGGFIWLSYIPRCWFS  HHHHHHH--EEEHHH-------  LEWFGDQSSGSSIRVPLPPHLYRATNWIGLAL  -----------EEEEE------------EEEH  FQWLPFGGFIWVSYIPRAWFS  EEEE-----EEEEE-------  LEWFGHQSNDSSATISLPHNLNLDSNWIGLAV  -----------EEEEE------------EEEE  FLWLHLGGFVWVSYIPRAWFSDQL  EEEEE----EEEEE---------- |
| **WW domain consensus in *Vitis vinifera*** | SDWQEHTSADGRRYYYNKKTRLSSWEKPLEL  --EEEEE-----EEEEE-----EEE------ |

**Additional file 5. Secondary structure prediction of the PL motif-1 & 2.** The table shows the consensus sequence obtained from the alignment of the peach PL sequences, the 5 PL of the Ma Protein and WW domain consensus sequence obtained from *Vitis vinifera* sequences. H signifies a helix residue. Predicted stands for extended beta sheet (E) are surrounded by the tryptophans (in blue).
