# Supplementary material for: TNL genes in peach: insights into the post-LRR domain
Source: BMC Genomics. 2016 Apr 30;17:317. doi: 10.1186/s12864-016-2635-0 (PMC4851768; doi:10.1186/s12864-016-2635-0)
Supplement: Additional file 7: — TNL-related peptides obtained with BLASTp analysis using MEME motifs of the post-LRR domain in the Plant Genome Database website. Results are shown for Populus trichocarpa, Vitis vinifera, Medicago truncatula, Arabidopsis thaliana, Solanum tuberosum, Glycine max, Oryza sativa, Prunus persica, Sorghum bicolor, and Zea mays. Identified TNL-related sequences, unrelated TNL sequences and unknown sequences are highlighted in green, blue and yellow, respectively. (DOC 138 kb) [file 12864_2016_2635_MOESM7_ESM.doc]

Database: *Populus Tricarpa* peptides

45,033 sequences; 17,278,601 total letters

Query= PL

Length=95

Score E

Sequences producing significant alignments: (Bits) Value

gnl|PtGDB|POPTR_0007s04750.1 PACid:18242427 93.2 1e-22 TNL

gnl|PtGDB|POPTR_0001s06610.1 PACid:18236132 60.8 2e-11

gnl|PtGDB|POPTR_1446s00200.1 PACid:18205974 59.3 5e-11

gnl|PtGDB|POPTR_0013s10630.2 PACid:18220891 54.3 2e-09

gnl|PtGDB|POPTR_0013s10630.1 PACid:18220890 54.3 2e-09

gnl|PtGDB|POPTR_0013s10700.1 PACid:18221016 53.5 5e-09

gnl|PtGDB|POPTR_0002s05700.1 PACid:18246116 52.4 1e-08

gnl|PtGDB|POPTR_0822s00200.1 PACid:18222079 51.6 2e-08

gnl|PtGDB|POPTR_0005s22800.1 PACid:18208836 50.8 3e-08

gnl|PtGDB|POPTR_0013s08870.1 PACid:18221672 50.4 4e-08

gnl|PtGDB|POPTR_0043s00200.1 PACid:18239493 50.8 4e-08

gnl|PtGDB|POPTR_0035s00260.1 PACid:18247343 50.1 7e-08

gnl|PtGDB|POPTR_0013s08850.1 PACid:18220771 49.3 1e-07

gnl|PtGDB|POPTR_0077s00230.1 PACid:18232497 47.8 4e-07

gnl|PtGDB|POPTR_0019s12930.1 PACid:18219046 47.8 4e-07

gnl|PtGDB|POPTR_0019s12990.1 PACid:18219770 47.4 5e-07

gnl|PtGDB|POPTR_0008s22430.1 PACid:18249803 47.4 5e-07

gnl|PtGDB|POPTR_0019s09730.1 PACid:18219806 47.4 5e-07

gnl|PtGDB|POPTR_0019s12920.1 PACid:18219322 47.4 5e-07

gnl|PtGDB|POPTR_0019s09860.1 PACid:18219263 47.4 6e-07

gnl|PtGDB|POPTR_0019s09880.1 PACid:18219014 47.4 6e-07

gnl|PtGDB|POPTR_0019s12570.1 PACid:18220060 47.0 6e-07

gnl|PtGDB|POPTR_0019s13030.1 PACid:18219744 47.0 7e-07

gnl|PtGDB|POPTR_0017s04350.1 PACid:18209838 47.0 8e-07

gnl|PtGDB|POPTR_0035s00200.1 PACid:18247331 46.2 9e-07

gnl|PtGDB|POPTR_0035s00380.1 PACid:18247325 46.2 1e-06

gnl|PtGDB|POPTR_0043s00240.1 PACid:18239491 46.2 1e-06

gnl|PtGDB|POPTR_0019s09840.1 PACid:18219775 45.8 2e-06

gnl|PtGDB|POPTR_0035s00341.1 PACid:18247322 45.4 2e-06

gnl|PtGDB|POPTR_0017s13730.1 PACid:18210042 45.4 2e-06

gnl|PtGDB|POPTR_0013s10670.1 PACid:18221351 44.7 3e-06

gnl|PtGDB|POPTR_0019s12770.1 PACid:18219992 44.7 3e-06

gnl|PtGDB|POPTR_0035s00400.1 PACid:18247334 45.1 3e-06

gnl|PtGDB|POPTR_0019s13010.1 PACid:18219555 45.1 3e-06

gnl|PtGDB|POPTR_0013s10740.1 PACid:18220873 44.7 4e-06

gnl|PtGDB|POPTR_0077s00240.1 PACid:18232496 44.7 4e-06

gnl|PtGDB|POPTR_0019s09760.1 PACid:18219158 43.1 1e-05

gnl|PtGDB|POPTR_0043s00306.1 PACid:18239488 42.7 2e-05

gnl|PtGDB|POPTR_0293s00200.1 PACid:18210853 42.0 2e-05

gnl|PtGDB|POPTR_0019s09620.1 PACid:18218944 42.0 3e-05

gnl|PtGDB|POPTR_0019s09820.1 PACid:18218855 41.6 4e-05

gnl|PtGDB|POPTR_0077s00200.1 PACid:18232499 39.7 1e-04

gnl|PtGDB|POPTR_0005s03190.1 PACid:18207878 38.9 2e-04

gnl|PtGDB|POPTR_0019s00920.1 PACid:18219085 38.9 2e-04

gnl|PtGDB|POPTR_0019s09630.1 PACid:18220051 37.7 6e-04

gnl|PtGDB|POPTR_0019s09795.1 PACid:18219004 37.0 9e-04

gnl|PtGDB|POPTR_0182s00240.1 PACid:18244073 37.4 9e-04

gnl|PtGDB|POPTR_0005s03350.1 PACid:18208774 37.4 0.001

gnl|PtGDB|POPTR_0019s09850.1 PACid:18219791 37.0 0.001

gnl|PtGDB|POPTR_0005s03020.1 PACid:18207586 36.2 0.002

gnl|PtGDB|POPTR_0005s03260.1 PACid:18208932 35.0 0.005

gnl|PtGDB|POPTR_0182s00200.1 PACid:18244074 33.9 0.011

gnl|PtGDB|POPTR_0019s00900.1 PACid:18219016 33.5 0.014

gnl|PtGDB|POPTR_0005s03360.1 PACid:18206029 33.5 0.018

gnl|PtGDB|POPTR_0005s03440.1 PACid:18207570 33.1 0.018

gnl|PtGDB|POPTR_0015s04000.1 PACid:18232920 33.1 0.024

gnl|PtGDB|POPTR_0003s08250.1 PACid:18218036 32.7 0.028

gnl|PtGDB|POPTR_0005s03090.1 PACid:18206819 32.3 0.029

gnl|PtGDB|POPTR_0005s03330.1 PACid:18206944 32.3 0.039

gnl|PtGDB|POPTR_0001s31480.1 PACid:18236333 30.8 0.14

gnl|PtGDB|POPTR_0548s00200.1 PACid:18251514 30.0 0.21

gnl|PtGDB|POPTR_0018s02985.1 PACid:18215362 30.0 0.22

gnl|PtGDB|POPTR_0017s14620.1 PACid:18210418 30.0 0.22

gnl|PtGDB|POPTR_0019s00950.1 PACid:18219201 30.0 0.26

gnl|PtGDB|POPTR_0005s03490.1 PACid:18207880 29.6 0.30

gnl|PtGDB|POPTR_0002s09650.1 PACid:18244704 29.3 0.48 sugar transporter

gnl|PtGDB|POPTR_0035s00400.2 PACid:18247335 28.1 1.0

gnl|PtGDB|POPTR_0003s06530.1 PACid:18218017 28.1 1.1 subtilase

gnl|PtGDB|POPTR_0007s00660.1 PACid:18242593 27.7 1.3 PL ?

gnl|PtGDB|POPTR_0014s12170.1 PACid:18222458 27.7 1.5 glycosyl hydrolase

gnl|PtGDB|POPTR_0013s08840.1 PACid:18220994 27.3 2.1

gnl|PtGDB|POPTR_0002s20350.1 PACid:18246797 26.9 2.6 glycosyl hydrolase

gnl|PtGDB|POPTR_0019s00870.1 PACid:18218981 26.6 3.8

gnl|PtGDB|POPTR_0001s16750.1 PACid:18235130 26.6 3.8 subtilase

gnl|PtGDB|POPTR_0019s00990.1 PACid:18218795 26.6 4.7

gnl|PtGDB|POPTR_0001s13900.1 PACid:18235993 25.8 6.6 acyl-CoA synthetase 5

gnl|PtGDB|POPTR_0006s23630.1 PACid:18212960 25.4 9.5 glycosyl hydrolase

Database: *Vitis vinifera* peptides

26,346 sequences; 9,986,113 total letters

Query= PL

Length=95

Score E

Sequences producing significant alignments: (Bits) Value

gnl|VvGDB|GSVIVT01026101001 assembled CDS 60.1 8e-12

gnl|VvGDB|GSVIVT01015681001 assembled CDS 54.3 2e-10

gnl|VvGDB|GSVIVT01004865001 assembled CDS 53.5 3e-09

gnl|VvGDB|GSVIVT01022979001 assembled CDS 52.0 8e-09

gnl|VvGDB|GSVIVT01012761001 assembled CDS 51.6 1e-08

gnl|VvGDB|GSVIVT01012759001 assembled CDS 49.3 5e-08

gnl|VvGDB|GSVIVT01034639001 assembled CDS 47.4 9e-08 PL

gnl|VvGDB|GSVIVT01004877001 assembled CDS 48.5 1e-07

gnl|VvGDB|GSVIVT01005725001 assembled CDS 47.4 2e-07

gnl|VvGDB|GSVIVT01025878001 assembled CDS 47.0 4e-07

gnl|VvGDB|GSVIVT01001785001 assembled CDS 45.4 5e-07

gnl|VvGDB|GSVIVT01012762001 assembled CDS 46.6 5e-07

gnl|VvGDB|GSVIVT01001797001 assembled CDS 45.8 7e-07

gnl|VvGDB|GSVIVT01004873001 assembled CDS 45.8 9e-07

gnl|VvGDB|GSVIVT01001801001 assembled CDS 45.1 1e-06

gnl|VvGDB|GSVIVT01005729001 assembled CDS 45.1 1e-06

gnl|VvGDB|GSVIVT01004867001 assembled CDS 41.2 3e-05

gnl|VvGDB|GSVIVT01025958001 assembled CDS 41.2 3e-05

gnl|VvGDB|GSVIVT01025875001 assembled CDS 40.8 3e-05

gnl|VvGDB|GSVIVT01025885001 assembled CDS 40.0 6e-05

gnl|VvGDB|GSVIVT01034809001 assembled CDS 39.3 1e-04

gnl|VvGDB|GSVIVT01034805001 assembled CDS 38.5 2e-04

gnl|VvGDB|GSVIVT01036401001 assembled CDS 37.0 7e-04

gnl|VvGDB|GSVIVT01025994001 assembled CDS 36.2 7e-04

gnl|VvGDB|GSVIVT01036409001 assembled CDS 36.6 8e-04

gnl|VvGDB|GSVIVT01001512001 assembled CDS 37.0 8e-04

gnl|VvGDB|GSVIVT01026003001 assembled CDS 36.6 9e-04

gnl|VvGDB|GSVIVT01036408001 assembled CDS 36.6 0.001

gnl|VvGDB|GSVIVT01000993001 assembled CDS 35.8 0.001

gnl|VvGDB|GSVIVT01037226001 assembled CDS 35.8 0.002

gnl|VvGDB|GSVIVT01034812001 assembled CDS 35.4 0.002

gnl|VvGDB|GSVIVT01001492001 assembled CDS 34.7 0.002

gnl|VvGDB|GSVIVT01029007001 assembled CDS 34.7 0.003

gnl|VvGDB|GSVIVT01029005001 assembled CDS 34.7 0.004

gnl|VvGDB|GSVIVT01025986001 assembled CDS 34.3 0.005

gnl|VvGDB|GSVIVT01020962001 assembled CDS 33.9 0.006

gnl|VvGDB|GSVIVT01019195001 assembled CDS 33.5 0.007

gnl|VvGDB|GSVIVT01025981001 assembled CDS 33.5 0.008

gnl|VvGDB|GSVIVT01037222001 assembled CDS 33.5 0.009 multiple PL

gnl|VvGDB|GSVIVT01025989001 assembled CDS 32.7 0.015

gnl|VvGDB|GSVIVT01037219001 assembled CDS 32.3 0.025 TNL PL LRR/Rmi PL LRR PL

gnl|VvGDB|GSVIVT01022982001 assembled CDS 31.2 0.031

gnl|VvGDB|GSVIVT01025997001 assembled CDS 32.0 0.032

gnl|VvGDB|GSVIVT01037218001 assembled CDS 31.6 0.035

gnl|VvGDB|GSVIVT01037203001 assembled CDS 31.2 0.054

gnl|VvGDB|GSVIVT01025918001 assembled CDS 30.4 0.12

gnl|VvGDB|GSVIVT01029013001 assembled CDS 30.0 0.13

gnl|VvGDB|GSVIVT01023025001 assembled CDS 30.0 0.13

gnl|VvGDB|GSVIVT01036294001 assembled CDS 30.0 0.14

gnl|VvGDB|GSVIVT01026098001 assembled CDS 29.6 0.20

gnl|VvGDB|GSVIVT01023035001 assembled CDS 28.9 0.27

gnl|VvGDB|GSVIVT01025955001 assembled CDS 28.9 0.31

gnl|VvGDB|GSVIVT01003611001 assembled CDS 28.9 0.32

gnl|VvGDB|GSVIVT01003613001 assembled CDS 27.3 1.1

gnl|VvGDB|GSVIVT01019664001 assembled CDS 26.9 1.7

gnl|VvGDB|GSVIVT01019196001 assembled CDS 26.6 2.3

gnl|VvGDB|GSVIVT01037181001 assembled CDS 26.2 2.7

gnl|VvGDB|GSVIVT01038755001 assembled CDS 26.2 2.8

gnl|VvGDB|GSVIVT01012535001 assembled CDS 26.2 2.9

gnl|VvGDB|GSVIVT01023047001 assembled CDS 26.2 3.0

gnl|VvGDB|GSVIVT01028042001 assembled CDS 26.2 3.2

gnl|VvGDB|GSVIVT01020123001 assembled CDS 25.8 4.7

gnl|VvGDB|GSVIVT01036440001 assembled CDS 25.8 4.8 sugar transporter

gnl|VvGDB|GSVIVT01033374001 assembled CDS 25.4 5.8 no apical meristem

gnl|VvGDB|GSVIVT01036439001 assembled CDS 25.4 6.6 sugar transporter

gnl|VvGDB|GSVIVT01012667001 assembled CDS 25.0 7.0 sugar transporter

gnl|VvGDB|GSVIVT01035481001 assembled CDS 25.0 7.3 glycosyl hydrolase

gnl|VvGDB|GSVIVT01023071001 assembled CDS 25.0 7.5 antimicrobial extrusion protein

gnl|VvGDB|GSVIVT01024198001 assembled CDS 24.6 9.7

Database: *Medicago Truncatula* peptides

47,529 sequences; 14,090,916 total letters

Query= PL1

Length=95

Score E

Sequences producing significant alignments: (Bits) Value

gnl|MtGDB|Medtr4g080660.1 NBS-LRR resistance-like protein (Fragm... 45.4 2e-06

gnl|MtGDB|Medtr6g087200.1 Elongation factor Ts (AHRD V1 *-*- B9R... 45.4 2e-06

gnl|MtGDB|Medtr4g080330.1 Tir-nbs-lrr resistance protein (Fragme... 45.4 2e-06

gnl|MtGDB|Medtr4g081270.1 Tir-nbs-lrr resistance protein (Fragme... 43.1 1e-05

gnl|MtGDB|Medtr2g039770.1 Tir-nbs-lrr resistance protein (AHRD V... 41.2 4e-05

gnl|MtGDB|Medtr6g088070.1 Tir-nbs-lrr resistance protein (Fragme... 41.2 5e-05

gnl|MtGDB|Medtr6g087260.1 Tir-nbs-lrr resistance protein (Fragme... 41.2 5e-05

gnl|MtGDB|Medtr4g080060.1 Tir-nbs-lrr resistance protein (AHRD V... 40.8 5e-05

gnl|MtGDB|Medtr5g047480.1 TMV resistance protein N (AHRD V1 ***-... 40.4 7e-05

gnl|MtGDB|Medtr4g080070.1 NBS resistance protein (AHRD V1 *-*- B... 40.0 1e-04

gnl|MtGDB|Medtr8g012080.1 NBS-containing resistance-like protein... 39.3 2e-04

gnl|MtGDB|Medtr4g080650.1 TIR-NBS-LRR type disease resistance pr... 39.3 2e-04

gnl|MtGDB|Medtr4g080320.1 TIR-NBS-LRR type disease resistance pr... 39.3 2e-04

gnl|MtGDB|Medtr3g044180.1 TIR-NBS-LRR type disease resistance pr... 39.3 2e-04

gnl|MtGDB|AC231371_3.1 TIR-NBS-LRR type disease resistance prote... 38.9 2e-04

gnl|MtGDB|Medtr5g047530.1 TMV resistance protein N (AHRD V1 ***-... 38.9 3e-04

gnl|MtGDB|Medtr6g027080.1 TIR-NBS-LRR type disease resistance pr... 38.1 3e-04

gnl|MtGDB|Medtr8g012180.1 NBS-containing resistance-like protein... 38.5 3e-04

gnl|MtGDB|Medtr4g080930.1 TIR-NBS-LRR type disease resistance pr... 38.1 4e-04

gnl|MtGDB|Medtr8g012000.1 Unknown Protein (AHRD V1) chr08_pseudo... 37.7 4e-04

gnl|MtGDB|Medtr8g011920.1 Unknown Protein (AHRD V1) chr08_pseudo... 37.7 4e-04

gnl|MtGDB|Medtr6g087220.1 Tir-nbs-lrr resistance protein (Fragme... 37.7 5e-04

gnl|MtGDB|Medtr2g083560.1 Tir-nbs-lrr resistance protein (AHRD V... 37.0 6e-04

gnl|MtGDB|Medtr7g025250.1 TMV resistance protein N (AHRD V1 ***-... 37.4 7e-04

gnl|MtGDB|Medtr2g083650.1 Disease-resistance protein (AHRD V1 *-... 37.4 7e-04

gnl|MtGDB|Medtr8g012200.1 TMV resistance protein N (AHRD V1 ***-... 37.0 0.001

gnl|MtGDB|Medtr6g084360.1 Tir-nbs-lrr resistance protein (AHRD V... 37.0 0.001

gnl|MtGDB|Medtr4g081220.1 Pentatricopeptide repeat-containing pr... 36.2 0.002

gnl|MtGDB|Medtr8g012190.1 TMV resistance protein N (AHRD V1 ***-... 35.8 0.002

gnl|MtGDB|Medtr6g087170.1 Tir-nbs-lrr resistance protein (Fragme... 35.8 0.003

gnl|MtGDB|Medtr5g036240.1 NBS-containing resistance-like protein... 35.4 0.003

gnl|MtGDB|AC233783_25.1 Elongation factor Ts (AHRD V1 *--- B9RKL... 35.4 0.003

gnl|MtGDB|Medtr2g083510.1 TIR-NBS-LRR-TIR type disease resistanc... 35.0 0.004

gnl|MtGDB|Medtr8g073730.1 TMV resistance protein N (AHRD V1 ***-... 34.7 0.005

gnl|MtGDB|Medtr4g014220.1 NBS-containing resistance-like protein... 34.7 0.006

gnl|MtGDB|Medtr4g081250.1 Tir-nbs-lrr resistance protein (Fragme... 34.3 0.007

gnl|MtGDB|Medtr7g055970.1 TMV resistance protein N (AHRD V1 ***-... 34.3 0.007

gnl|MtGDB|Medtr8g011850.1 TMV resistance protein N (AHRD V1 ***-... 34.3 0.007

gnl|MtGDB|Medtr3g019080.1 Resistance protein (Fragment) (AHRD V1... 34.3 0.008

gnl|MtGDB|Medtr7g088950.1 TMV resistance protein N (AHRD V1 *-*-... 33.9 0.010

gnl|MtGDB|Medtr7g021420.1 TMV resistance protein N (AHRD V1 ***-... 33.5 0.010

gnl|MtGDB|Medtr3g072140.1 TMV resistance protein N (AHRD V1 ***-... 33.5 0.011

gnl|MtGDB|Medtr4g014320.1 Tir-nbs-lrr resistance protein (Fragme... 33.5 0.012

gnl|MtGDB|Medtr4g043630.1 NBS-LRR resistance-like protein 4G (Fr... 33.5 0.014

gnl|MtGDB|Medtr8g038820.1 NBS-LRR resistance-like protein 4G (Fr... 33.5 0.014

gnl|MtGDB|Medtr6g088250.1 Elongation factor Ts (AHRD V1 *-*- B9R... 33.5 0.014

gnl|MtGDB|Medtr6g087320.1 Tir-nbs-lrr resistance protein (AHRD V... 33.5 0.015

gnl|MtGDB|Medtr5g029940.1 Unknown Protein (AHRD V1) chr05_pseudo... 32.7 0.015

gnl|MtGDB|Medtr4g013820.1 NBS-containing resistance-like protein... 33.1 0.018

gnl|MtGDB|AC235669_3.1 Cellulose synthase (Fragment) (AHRD V1 *-... 33.1 0.021

gnl|MtGDB|Medtr8g011910.1 Resistance protein MG55 (Fragment) (AH... 32.7 0.022

gnl|MtGDB|Medtr8g039910.1 Disease resistance-like protein GS3-1 ... 32.7 0.024

gnl|MtGDB|Medtr8g039870.1 Disease resistance-like protein GS3-1 ... 32.7 0.024

gnl|MtGDB|Medtr4g015060.1 TIR-NBS-LRR RCT1-like resistance prote... 32.7 0.025

gnl|MtGDB|Medtr6g081080.1 Nbs-lrr resistance protein (AHRD V1 *-... 32.3 0.028

gnl|MtGDB|Medtr8g020430.1 Resistance protein (Fragment) (AHRD V1... 32.0 0.037

gnl|MtGDB|Medtr4g081330.1 Tir-nbs-lrr resistance protein (Fragme... 32.0 0.039

gnl|MtGDB|Medtr7g078180.1 TMV resistance protein N (AHRD V1 ***-... 32.0 0.045

gnl|MtGDB|Medtr2g083520.1 TIR-NBS-LRR type disease resistance pr... 32.0 0.046

gnl|MtGDB|Medtr2g099920.1 Tir-nbs-lrr resistance protein (AHRD V... 32.0 0.048

gnl|MtGDB|Medtr4g014570.1 Disease resistance protein (AHRD V1 **... 31.6 0.052

gnl|MtGDB|Medtr3g022930.1 TMV resistance protein N (AHRD V1 *-*-... 31.6 0.052

gnl|MtGDB|Medtr3g104210.1 TIR-NBS-LRR RCT1-like resistance prote... 31.2 0.053

gnl|MtGDB|Medtr3g104130.1 TIR-NBS-LRR RCT1-like resistance prote... 31.2 0.053

gnl|MtGDB|Medtr3g104240.1 TIR-NBS-LRR RCT1-like resistance prote... 31.2 0.053

gnl|MtGDB|Medtr3g104170.1 TIR-NBS-LRR RCT1-like resistance prote... 31.2 0.053

gnl|MtGDB|Medtr7g078780.1 TMV resistance protein N (AHRD V1 ***-... 31.6 0.054

gnl|MtGDB|Medtr4g014340.1 Tir-nbs-lrr resistance protein (Fragme... 31.6 0.055

gnl|MtGDB|Medtr4g014240.1 Tir-nbs-lrr resistance protein (Fragme... 31.6 0.062

gnl|MtGDB|Medtr8g086780.1 Resistance protein (Fragment) (AHRD V1... 31.2 0.070

gnl|MtGDB|Medtr8g087130.1 Resistance protein (Fragment) (AHRD V1... 31.2 0.071

gnl|MtGDB|Medtr4g080080.1 NBS-LRR resistance-like protein 4U (Fr... 31.2 0.072

gnl|MtGDB|Medtr4g014210.1 Unknown Protein (AHRD V1) chr04_pseudo... 30.8 0.088

gnl|MtGDB|Medtr6g087850.1 Tir-nbs-lrr resistance protein (AHRD V... 31.2 0.090

gnl|MtGDB|Medtr4g014980.1 TIR-NBS-LRR RCT1 resistance protein (A... 30.4 0.11

gnl|MtGDB|Medtr2g066640.1 Unknown Protein (AHRD V1) chr02_pseudo... 30.4 0.11

gnl|MtGDB|Medtr8g018000.1 Disease resistance protein (Fragment) ... 30.8 0.12

gnl|MtGDB|Medtr7g038520.1 TMV resistance protein N (AHRD V1 **--... 30.4 0.16

gnl|MtGDB|Medtr4g015030.1 TIR-NBS-LRR RCT1-like resistance prote... 30.0 0.19

gnl|MtGDB|Medtr4g081290.1 NBS-LRR resistance-like protein 4F (Fr... 30.0 0.22

gnl|MtGDB|Medtr4g014310.1 NBS-containing resistance-like protein... 29.6 0.25

gnl|MtGDB|Medtr2g037690.1 TMV resistance protein N (AHRD V1 ***-... 29.6 0.26

gnl|MtGDB|Medtr4g081230.1 Tir-nbs-lrr resistance protein (Fragme... 29.6 0.26

gnl|MtGDB|Medtr6g088260.1 Tir-nbs-lrr resistance protein (Fragme... 29.6 0.29

gnl|MtGDB|Medtr4g023400.1 TMV resistance protein N (AHRD V1 *-*-... 29.6 0.31

gnl|MtGDB|Medtr4g014120.1 NBS-containing resistance-like protein... 28.9 0.42

gnl|MtGDB|Medtr2g099860.1 Unknown Protein (AHRD V1) chr02_pseudo... 28.5 0.48

gnl|MtGDB|Medtr8g104820.1 Endo-beta-1 4-glucanase (AHRD V1 ***- ... 28.9 0.55

gnl|MtGDB|Medtr4g015050.1 TIR-NBS-LRR RCT1-like resistance prote... 28.5 0.64

gnl|MtGDB|Medtr6g072980.1 Unknown Protein (AHRD V1) chr06_pseudo... 27.7 0.77

gnl|MtGDB|Medtr3g079790.1 TIR-NBS-LRR type disease resistance pr... 28.1 0.84

gnl|MtGDB|Medtr4g014280.1 Tir-nbs-lrr resistance protein (Fragme... 28.1 1.0

gnl|MtGDB|Medtr1g104550.1 Primary amine oxidase (AHRD V1 *--- B9... 28.1 1.1

gnl|MtGDB|Medtr3g079780.1 Tir-nbs-lrr resistance protein (Fragme... 28.1 1.1

gnl|MtGDB|Medtr6g079120.1 Resistance protein (AHRD V1 ***- C6ZS3... 27.7 1.1

gnl|MtGDB|Medtr8g020300.1 NBS-containing resistance-like protein... 27.7 1.2

gnl|MtGDB|Medtr5g066530.1 Sentrin-specific protease 6 (AHRD V1 *... 27.3 1.5

gnl|MtGDB|Medtr6g071550.1 Disease resistance protein (AHRD V1 **... 27.3 1.8

gnl|MtGDB|Medtr8g018170.1 Disease resistance protein (Fragment) ... 27.3 1.8

gnl|MtGDB|Medtr7g078270.1 Tir-nbs-lrr resistance protein (AHRD V... 27.3 1.9

gnl|MtGDB|Medtr6g077640.1 Resistance protein (Fragment) (AHRD V1... 26.9 2.4

gnl|MtGDB|Medtr4g068200.1 Tir-nbs-lrr resistance protein (Fragme... 26.9 2.5

gnl|MtGDB|Medtr6g078420.1 NBS-containing resistance-like protein... 26.9 2.5

gnl|MtGDB|Medtr8g011950.1 Resistance protein (Fragment) (AHRD V1... 26.6 3.8

gnl|MtGDB|Medtr4g124590.1 1-phosphatidylinositol-3-phosphate 5-k... 26.2 4.0

gnl|MtGDB|Medtr8g103910.1 Tir-nbs-lrr resistance protein (AHRD V... 26.2 4.0

gnl|MtGDB|Medtr6g074810.1 Disease resistance-like protein GS3-3 ... 25.8 6.0

gnl|MtGDB|Medtr8g011870.1 Unknown Protein (AHRD V1) chr08_pseudo... 25.8 6.1

gnl|MtGDB|Medtr8g017800.1 Disease resistance protein (Fragment) ... 25.8 6.4

gnl|MtGDB|Medtr5g040490.1 Disease resistance-like protein (Fragm... 25.8 7.0

gnl|MtGDB|Medtr8g017970.1 Tir-nbs-lrr resistance protein (AHRD V... 25.4 7.5

gnl|MtGDB|Medtr6g075880.1 Disease resistance-like protein (Fragm... 25.4 8.5

gnl|MtGDB|Medtr8g067400.1 Endo-1 4-beta-glucanase (Fragment) (AH... 25.4 8.7

gnl|MtGDB|Medtr3g080810.1 Endo-1 4-beta-glucanase (Fragment) (AH... 25.4 8.7

Database: *Arabidopsis thaliana* peptides

35,386 sequences; 14,518,241 total letters

Query= PL1

Length=95

Score E

Sequences producing significant alignments: (Bits) Value

gnl|AtGDB|AT3G51570.1 | Symbols: | Disease resistance protein (... 49.3 1e-07

gnl|AtGDB|AT4G16950.2 | Symbols: RPP5 | Disease resistance prote... 48.5 2e-07

gnl|AtGDB|AT4G19470.1 | Symbols: | Leucine-rich repeat (LRR) fa... 47.8 2e-07

gnl|AtGDB|AT5G45230.1 | Symbols: | Disease resistance protein (... 48.5 2e-07

gnl|AtGDB|AT5G45250.1 | Symbols: RPS4 | Disease resistance prote... 48.1 3e-07

gnl|AtGDB|AT4G19530.1 | Symbols: | disease resistance protein (... 46.6 8e-07

gnl|AtGDB|AT5G45200.1 | Symbols: | Disease resistance protein (... 44.3 4e-06

gnl|AtGDB|AT5G17880.1 | Symbols: CSA1 | disease resistance prote... 44.3 5e-06

gnl|AtGDB|AT5G45060.1 | Symbols: | Disease resistance protein (... 43.5 8e-06

gnl|AtGDB|AT4G36150.1 | Symbols: | Disease resistance protein (... 41.2 5e-05

gnl|AtGDB|AT4G16950.1 | Symbols: RPP5 | Disease resistance prote... 41.2 5e-05

gnl|AtGDB|AT4G12010.1 | Symbols: | Disease resistance protein (... 38.5 3e-04

gnl|AtGDB|AT2G17060.1 | Symbols: | Disease resistance protein (... 38.1 4e-04

gnl|AtGDB|AT4G19510.1 | Symbols: | Disease resistance protein (... 38.1 4e-04

gnl|AtGDB|AT1G63750.2 | Symbols: | Disease resistance protein (... 35.8 0.002

gnl|AtGDB|AT1G63750.1 | Symbols: | Disease resistance protein (... 35.8 0.002

gnl|AtGDB|AT1G63750.3 | Symbols: | Disease resistance protein (... 35.8 0.002

gnl|AtGDB|AT5G40060.1 | Symbols: | Disease resistance protein (... 35.0 0.004

gnl|AtGDB|AT4G19510.2 | Symbols: | Disease resistance protein (... 35.0 0.004

gnl|AtGDB|AT5G44870.1 | Symbols: LAZ5 | Disease resistance prote... 34.7 0.005

gnl|AtGDB|AT5G41550.1 | Symbols: | Disease resistance protein (... 34.7 0.006

gnl|AtGDB|AT5G18370.1 | Symbols: | Disease resistance protein (... 34.3 0.008

gnl|AtGDB|AT1G63740.1 | Symbols: | Disease resistance protein (... 33.9 0.010

gnl|AtGDB|AT5G46470.1 | Symbols: RPS6 | disease resistance prote... 33.9 0.010

gnl|AtGDB|AT5G44510.1 | Symbols: TAO1 | target of AVRB operation... 33.9 0.010

gnl|AtGDB|AT5G11250.1 | Symbols: | Disease resistance protein (... 33.9 0.011

gnl|AtGDB|AT4G08450.1 | Symbols: | Disease resistance protein (... 32.7 0.023

gnl|AtGDB|AT5G41540.1 | Symbols: | Disease resistance protein (... 32.7 0.023

gnl|AtGDB|AT3G48770.1 | Symbols: | DNA binding;ATP binding | ch... 32.7 0.024

gnl|AtGDB|AT4G11170.1 | Symbols: | Disease resistance protein (... 32.3 0.030

gnl|AtGDB|AT5G40910.1 | Symbols: | Disease resistance protein (... 32.3 0.033

gnl|AtGDB|AT1G63880.1 | Symbols: | Disease resistance protein (... 31.2 0.079

gnl|AtGDB|AT5G49140.1 | Symbols: | Disease resistance protein (... 31.2 0.080

gnl|AtGDB|AT5G17680.1 | Symbols: | disease resistance protein (... 30.4 0.14

gnl|AtGDB|AT5G58120.1 | Symbols: | Disease resistance protein (... 30.4 0.14

gnl|AtGDB|AT1G57650.2 | Symbols: | ATP binding | chr1:21351291-... 30.4 0.14

gnl|AtGDB|AT1G56510.1 | Symbols: WRR4, ADR2 | Disease resistance... 30.0 0.18

gnl|AtGDB|AT1G63860.2 | Symbols: | Disease resistance protein (... 30.0 0.23

gnl|AtGDB|AT1G63860.1 | Symbols: | Disease resistance protein (... 30.0 0.23

gnl|AtGDB|AT5G41750.2 | Symbols: | Disease resistance protein (... 29.6 0.24

gnl|AtGDB|AT5G41750.1 | Symbols: | Disease resistance protein (... 29.6 0.24

gnl|AtGDB|AT1G56540.1 | Symbols: | Disease resistance protein (... 29.6 0.24

gnl|AtGDB|AT5G38850.1 | Symbols: | Disease resistance protein (... 29.6 0.31

gnl|AtGDB|AT5G46270.1 | Symbols: | Disease resistance protein (... 29.3 0.32

gnl|AtGDB|AT1G57650.1 | Symbols: | ATP binding | chr1:21351291-... 29.3 0.32

gnl|AtGDB|AT3G25510.1 | Symbols: | disease resistance protein (... 29.3 0.33

gnl|AtGDB|AT4G14370.1 | Symbols: | Disease resistance protein (... 29.3 0.35

gnl|AtGDB|AT1G63730.1 | Symbols: | Disease resistance protein (... 29.3 0.40

gnl|AtGDB|AT3G44670.2 | Symbols: | Disease resistance protein (... 28.9 0.53

gnl|AtGDB|AT3G44670.1 | Symbols: | Disease resistance protein (... 28.9 0.53

gnl|AtGDB|AT5G38340.1 | Symbols: | Disease resistance protein (... 28.9 0.58

gnl|AtGDB|AT5G38350.1 | Symbols: | Disease resistance protein (... 28.5 0.74

gnl|AtGDB|AT1G65850.2 | Symbols: | Disease resistance protein (... 28.1 1.0

gnl|AtGDB|AT1G65850.1 | Symbols: | Disease resistance protein (... 28.1 1.0

gnl|AtGDB|AT5G46510.1 | Symbols: | Disease resistance protein (... 27.7 1.3

gnl|AtGDB|AT5G46520.1 | Symbols: | Disease resistance protein (... 27.7 1.4

gnl|AtGDB|AT1G27170.2 | Symbols: | transmembrane receptors;ATP ... 27.3 1.7

gnl|AtGDB|AT1G27170.1 | Symbols: | transmembrane receptors;ATP ... 27.3 1.7

gnl|AtGDB|AT1G27180.1 | Symbols: | disease resistance protein (... 27.3 1.7

gnl|AtGDB|AT1G11260.1 | Symbols: STP1, ATSTP1 | sugar transporte... 27.3 1.7

gnl|AtGDB|AT5G41740.1 | Symbols: | Disease resistance protein (... 27.3 1.7

gnl|AtGDB|AT5G41740.2 | Symbols: | Disease resistance protein (... 27.3 1.7

gnl|AtGDB|AT1G69550.1 | Symbols: | disease resistance protein (... 27.3 2.0

gnl|AtGDB|AT5G51630.2 | Symbols: | Disease resistance protein (... 26.9 2.5

gnl|AtGDB|AT5G51630.3 | Symbols: | Disease resistance protein (... 26.9 2.5

gnl|AtGDB|AT5G51630.1 | Symbols: | Disease resistance protein (... 26.9 2.7

gnl|AtGDB|AT5G22690.1 | Symbols: | Disease resistance protein (... 26.9 2.8

gnl|AtGDB|AT4G02290.1 | Symbols: AtGH9B13, GH9B13 | glycosyl hyd... 26.6 3.3

gnl|AtGDB|AT4G21480.1 | Symbols: STP12 | sugar transporter prote... 26.6 3.5

gnl|AtGDB|AT4G39000.1 | Symbols: AtGH9B17, GH9B17 | glycosyl hyd... 26.6 3.8

gnl|AtGDB|AT4G16920.1 | Symbols: | Disease resistance protein (... 26.2 4.9

gnl|AtGDB|AT4G16890.1 | Symbols: SNC1, BAL | disease resistance ... 25.4 7.8

Database: *Solanum tuberosum* peptides

56,218 sequences; 16,952,062 total letters

Query= PL1

Length=95

Score E

Sequences producing significant alignments: (Bits) Value

gnl|StGDB|PGSC0003DMP400023190 PGSC0003DMT400034089 67.4 8e-14

gnl|StGDB|PGSC0003DMP400031726 PGSC0003DMT400046894 67.8 8e-14

gnl|StGDB|PGSC0003DMP400023192 PGSC0003DMT400034091 67.4 1e-13

gnl|StGDB|PGSC0003DMP400023191 PGSC0003DMT400034090 67.4 1e-13

gnl|StGDB|PGSC0003DMP400031802 PGSC0003DMT400047000 65.5 5e-13

gnl|StGDB|PGSC0003DMP400031803 PGSC0003DMT400047001 65.5 5e-13

gnl|StGDB|PGSC0003DMP400023183 PGSC0003DMT400034074 60.8 2e-11

gnl|StGDB|PGSC0003DMP400036041 PGSC0003DMT400053416 56.6 4e-10

gnl|StGDB|PGSC0003DMP400036040 PGSC0003DMT400053415 56.6 5e-10

gnl|StGDB|PGSC0003DMP400010670 PGSC0003DMT400015397 54.3 3e-09

gnl|StGDB|PGSC0003DMP400018620 PGSC0003DMT400027297 52.8 3e-09

gnl|StGDB|PGSC0003DMP400036042 PGSC0003DMT400053417 46.6 2e-07

gnl|StGDB|PGSC0003DMP400029681 PGSC0003DMT400043741 47.4 3e-07

gnl|StGDB|PGSC0003DMP400036044 PGSC0003DMT400053419 47.0 7e-07

gnl|StGDB|PGSC0003DMP400036043 PGSC0003DMT400053418 47.0 8e-07

gnl|StGDB|PGSC0003DMP400024105 PGSC0003DMT400035453 45.8 2e-06

gnl|StGDB|PGSC0003DMP400030257 PGSC0003DMT400044611 44.7 5e-06

gnl|StGDB|PGSC0003DMP400045224 PGSC0003DMT400067053 42.0 2e-05

gnl|StGDB|PGSC0003DMP400045223 PGSC0003DMT400067052 42.0 2e-05

gnl|StGDB|PGSC0003DMP400032156 PGSC0003DMT400047499 42.0 3e-05

gnl|StGDB|PGSC0003DMP400005063 PGSC0003DMT400007274 40.8 8e-05

gnl|StGDB|PGSC0003DMP400019381 PGSC0003DMT400028484 40.4 8e-05

gnl|StGDB|PGSC0003DMP400019380 PGSC0003DMT400028483 40.4 8e-05

gnl|StGDB|PGSC0003DMP400053466 PGSC0003DMT400078881 39.3 2e-04

gnl|StGDB|PGSC0003DMP400053468 PGSC0003DMT400078883 39.3 2e-04

gnl|StGDB|PGSC0003DMP400001685 PGSC0003DMT400002320 38.9 3e-04

gnl|StGDB|PGSC0003DMP400054564 PGSC0003DMT400080445 38.5 3e-04

gnl|StGDB|PGSC0003DMP400029367 PGSC0003DMT400043302 38.5 4e-04

gnl|StGDB|PGSC0003DMP400028988 PGSC0003DMT400042719 37.4 6e-04

gnl|StGDB|PGSC0003DMP400027490 PGSC0003DMT400040535 37.7 6e-04

gnl|StGDB|PGSC0003DMP400004308 PGSC0003DMT400006232 37.7 6e-04

gnl|StGDB|PGSC0003DMP400004307 PGSC0003DMT400006231 37.7 6e-04

gnl|StGDB|PGSC0003DMP400030194 PGSC0003DMT400044516 37.0 0.001

gnl|StGDB|PGSC0003DMP400029366 PGSC0003DMT400043301 35.4 0.004

gnl|StGDB|PGSC0003DMP400029365 PGSC0003DMT400043300 35.4 0.004

gnl|StGDB|PGSC0003DMP400029683 PGSC0003DMT400043748 35.0 0.004

gnl|StGDB|PGSC0003DMP400039354 PGSC0003DMT400058450 34.7 0.006

gnl|StGDB|PGSC0003DMP400029678 PGSC0003DMT400043733 33.9 0.011

gnl|StGDB|PGSC0003DMP400054789 PGSC0003DMT400080824 33.5 0.013

gnl|StGDB|PGSC0003DMP400052261 PGSC0003DMT400077129 33.5 0.013

gnl|StGDB|PGSC0003DMP400052260 PGSC0003DMT400077128 33.5 0.013

gnl|StGDB|PGSC0003DMP400029677 PGSC0003DMT400043732 33.5 0.017

gnl|StGDB|PGSC0003DMP400032105 PGSC0003DMT400047418 33.1 0.022

gnl|StGDB|PGSC0003DMP400056180 PGSC0003DMT400083945 32.3 0.031

gnl|StGDB|PGSC0003DMP400056179 PGSC0003DMT400083944 32.3 0.031

gnl|StGDB|PGSC0003DMP400027528 PGSC0003DMT400040590 32.3 0.039

gnl|StGDB|PGSC0003DMP400004309 PGSC0003DMT400006233 32.0 0.048

gnl|StGDB|PGSC0003DMP400054787 PGSC0003DMT400080813 32.0 0.053

gnl|StGDB|PGSC0003DMP400048338 PGSC0003DMT400071445 32.0 0.058

gnl|StGDB|PGSC0003DMP400037912 PGSC0003DMT400056338 31.6 0.076

gnl|StGDB|PGSC0003DMP400037911 PGSC0003DMT400056337 31.6 0.076

gnl|StGDB|PGSC0003DMP400032104 PGSC0003DMT400047416 31.6 0.078

gnl|StGDB|PGSC0003DMP400055978 PGSC0003DMT400083471 30.0 0.20

gnl|StGDB|PGSC0003DMP400055979 PGSC0003DMT400083472 30.0 0.21

gnl|StGDB|PGSC0003DMP400055022 PGSC0003DMT400081314 30.0 0.21

gnl|StGDB|PGSC0003DMP400026519 PGSC0003DMT400039057 30.0 0.24

gnl|StGDB|PGSC0003DMP400055977 PGSC0003DMT400083470 30.0 0.25

gnl|StGDB|PGSC0003DMP400055976 PGSC0003DMT400083469 29.6 0.26

gnl|StGDB|PGSC0003DMP400048564 PGSC0003DMT400071797 29.3 0.34

gnl|StGDB|PGSC0003DMP400045902 PGSC0003DMT400067963 28.1 1.0

gnl|StGDB|PGSC0003DMP400016353 PGSC0003DMT400023974 27.7 1.1

gnl|StGDB|PGSC0003DMP400045906 PGSC0003DMT400067974 28.1 1.1

gnl|StGDB|PGSC0003DMP400045905 PGSC0003DMT400067973 28.1 1.1

gnl|StGDB|PGSC0003DMP400036348 PGSC0003DMT400053954 28.1 1.1

gnl|StGDB|PGSC0003DMP400032151 PGSC0003DMT400047494 28.1 1.2

gnl|StGDB|PGSC0003DMP400045907 PGSC0003DMT400067975 27.3 1.7

gnl|StGDB|PGSC0003DMP400045335 PGSC0003DMT400067204 26.6 3.7

gnl|StGDB|PGSC0003DMP400045334 PGSC0003DMT400067203 26.6 3.7

gnl|StGDB|PGSC0003DMP400026556 PGSC0003DMT400039127 26.2 4.4

gnl|StGDB|PGSC0003DMP400028583 PGSC0003DMT400042165 26.2 4.6

gnl|StGDB|PGSC0003DMP400032866 PGSC0003DMT400048535 26.2 4.6

gnl|StGDB|PGSC0003DMP400021647 PGSC0003DMT400031897 26.2 4.8

gnl|StGDB|PGSC0003DMP400021646 PGSC0003DMT400031896 26.2 5.0

gnl|StGDB|PGSC0003DMP400021534 PGSC0003DMT400031762 26.2 6.1

gnl|StGDB|PGSC0003DMP400030678 PGSC0003DMT400045277 25.8 7.4

Database: *Glycin max* peptides

75,778 sequences; 25,492,647 total letters

Query= PL1

Length=95

Score E

Sequences producing significant alignments: (Bits) Value

gnl|GmGDB|Glyma12g15830.2 unnamed protein product 43.5 1e-05 disease resistance protein (TIR-NBS-LRR class)

gnl|GmGDB|Glyma06g43850.1 unnamed protein product 43.5 1e-05

gnl|GmGDB|Glyma12g15860.2 unnamed protein product 41.6 5e-05

gnl|GmGDB|Glyma16g10270.1 unnamed protein product 39.7 3e-04

gnl|GmGDB|Glyma06g40740.1 unnamed protein product 39.3 4e-04

gnl|GmGDB|Glyma06g40710.1 unnamed protein product 38.5 7e-04

gnl|GmGDB|Glyma06g40950.1 unnamed protein product 38.1 7e-04

gnl|GmGDB|Glyma06g40980.1 unnamed protein product 38.1 8e-04

gnl|GmGDB|Glyma16g09950.1 unnamed protein product 36.6 8e-04

gnl|GmGDB|Glyma06g40690.1 unnamed protein product 38.1 8e-04

gnl|GmGDB|Glyma06g42730.1 unnamed protein product 37.0 0.002

gnl|GmGDB|Glyma13g42510.1 unnamed protein product 36.6 0.002

gnl|GmGDB|Glyma15g02870.1 unnamed protein product 37.0 0.002

gnl|GmGDB|Glyma01g03960.1 unnamed protein product 36.2 0.003

gnl|GmGDB|Glyma16g10020.1 unnamed protein product 35.8 0.004

gnl|GmGDB|Glyma12g15960.1 unnamed protein product 35.8 0.004

gnl|GmGDB|Glyma03g22060.1 unnamed protein product 33.1 0.035

gnl|GmGDB|Glyma06g39960.1 unnamed protein product 33.1 0.035

gnl|GmGDB|Glyma06g41380.1 unnamed protein product 33.1 0.037

gnl|GmGDB|Glyma08g20350.1 unnamed protein product 32.7 0.038

gnl|GmGDB|Glyma01g03980.1 unnamed protein product 32.7 0.041

gnl|GmGDB|Glyma06g40780.1 unnamed protein product 32.7 0.042

gnl|GmGDB|Glyma19g02670.1 unnamed protein product 32.3 0.056

gnl|GmGDB|Glyma01g03920.1 unnamed protein product 32.0 0.079

gnl|GmGDB|Glyma12g15850.1 unnamed protein product 32.0 0.086

gnl|GmGDB|Glyma03g14890.1 unnamed protein product 30.4 0.23 unknown

gnl|GmGDB|Glyma16g03780.1 unnamed protein product 30.4 0.28

gnl|GmGDB|Glyma03g06870.1 unnamed protein product 30.0 0.32

gnl|GmGDB|Glyma03g05950.1 unnamed protein product 30.0 0.33

gnl|GmGDB|Glyma19g07700.2 unnamed protein product 30.0 0.39

gnl|GmGDB|Glyma19g07680.1 unnamed protein product 30.0 0.40

gnl|GmGDB|Glyma13g26460.2 unnamed protein product 29.6 0.49

gnl|GmGDB|Glyma13g26460.1 unnamed protein product 29.6 0.49

gnl|GmGDB|Glyma19g07700.1 unnamed protein product 29.6 0.51

gnl|GmGDB|Glyma13g26420.1 unnamed protein product 29.6 0.52

gnl|GmGDB|Glyma01g31550.1 unnamed protein product 29.3 0.57

gnl|GmGDB|Glyma08g41270.1 unnamed protein product 29.3 0.59

gnl|GmGDB|Glyma06g41290.1 unnamed protein product 29.3 0.72

gnl|GmGDB|Glyma03g37420.1 unnamed protein product 28.9 0.95 glycosyl hydrolase

gnl|GmGDB|Glyma16g10080.1 unnamed protein product 28.5 1.3

gnl|GmGDB|Glyma16g33640.1 unnamed protein product 28.1 1.4

gnl|GmGDB|Glyma09g08850.1 unnamed protein product 28.1 1.5

gnl|GmGDB|Glyma19g40030.1 unnamed protein product 27.7 1.9 glycosyl hydrolase

gnl|GmGDB|Glyma01g04000.1 unnamed protein product 27.7 2.2

gnl|GmGDB|Glyma12g16450.1 unnamed protein product 27.7 2.4

gnl|GmGDB|Glyma06g46660.1 unnamed protein product 27.7 2.5

gnl|GmGDB|Glyma13g17510.1 unnamed protein product 27.7 2.7

gnl|GmGDB|Glyma01g27440.1 unnamed protein product 27.3 3.2

gnl|GmGDB|Glyma16g25020.1 unnamed protein product 27.3 3.4

gnl|GmGDB|Glyma16g33780.1 unnamed protein product 27.3 3.4

gnl|GmGDB|Glyma04g11130.1 unnamed protein product 27.3 3.5 monosaccharide transporter

gnl|GmGDB|Glyma16g33590.1 unnamed protein product 26.9 3.9

gnl|GmGDB|Glyma06g40680.1 unnamed protein product 26.9 4.0 unknown

gnl|GmGDB|Glyma07g31620.1 unnamed protein product 26.6 5.7 pentatricopeptide (PPR)

gnl|GmGDB|Glyma16g33910.2 unnamed protein product 26.6 6.4

gnl|GmGDB|Glyma16g33910.1 unnamed protein product 26.6 6.7

gnl|GmGDB|Glyma08g04840.1 unnamed protein product 26.2 7.5 glycosyl hydrolase

gnl|GmGDB|Glyma05g34850.1 unnamed protein product 26.2 7.5 glycosyl hydrolase

Database: *Oriza Sativa* peptides

66,338 sequences; 29,675,462 total letters

Query= PL1

Length=95

Score E

Sequences producing significant alignments: (Bits) Value

gnl|OsGDB|LOC_Os01g38680.1 PACid:21905524 28.1 1.7 sugar transporter 1

gnl|OsGDB|LOC_Os01g38670.1 PACid:21905570 28.1 1.8 sugar transporter 1

gnl|OsGDB|LOC_Os04g38220.1 PACid:21894107 27.3 3.2 Major facilitator

gnl|OsGDB|LOC_Os02g16270.2 PACid:21922107 26.2 9.8 NB-ARC domain-containing disease resistance protein

gnl|OsGDB|LOC_Os02g16270.1 PACid:21922106 26.2 9.8 NB-ARC domain-containing disease resistance protein

Database: *Prunus persica* peptides

28,689 sequences; 11,580,753 total letters

Query= PL1

Length=95

Score E

Sequences producing significant alignments: (Bits) Value

gnl|PeGDB|ppa000551m unnamed protein product 87.8 7e-21

gnl|PeGDB|ppa022023m unnamed protein product 85.5 4e-20

gnl|PeGDB|ppa026786m unnamed protein product 84.7 8e-20

gnl|PeGDB|ppa020601m unnamed protein product 82.8 1e-19

gnl|PeGDB|ppa000891m unnamed protein product 83.2 2e-19

gnl|PeGDB|ppa000525m unnamed protein product 82.4 6e-19

gnl|PeGDB|ppa023828m unnamed protein product 82.0 7e-19

gnl|PeGDB|ppa012913m unnamed protein product 77.4 8e-19 PL alone

gnl|PeGDB|ppa024258m unnamed protein product 81.3 1e-18

gnl|PeGDB|ppa017433m unnamed protein product 81.3 1e-18

gnl|PeGDB|ppa024462m unnamed protein product 80.9 2e-18

gnl|PeGDB|ppa000524m unnamed protein product 80.5 2e-18

gnl|PeGDB|ppa000477m unnamed protein product 79.7 4e-18

gnl|PeGDB|ppa026003m unnamed protein product 79.0 6e-18

gnl|PeGDB|ppa017291m unnamed protein product 78.6 1e-17

gnl|PeGDB|ppa024626m unnamed protein product 77.8 2e-17

gnl|PeGDB|ppa025473m unnamed protein product 76.6 4e-17

gnl|PeGDB|ppa023276m unnamed protein product 75.5 1e-16

gnl|PeGDB|ppa015313m unnamed protein product 74.7 2e-16

gnl|PeGDB|ppa017276m unnamed protein product 73.2 7e-16 TNL with PL new finding

gnl|PeGDB|ppa025931m unnamed protein product 72.8 1e-15

gnl|PeGDB|ppa015500m unnamed protein product 72.0 2e-15

gnl|PeGDB|ppa024336m unnamed protein product 71.2 4e-15

gnl|PeGDB|ppa018765m unnamed protein product 70.9 6e-15

gnl|PeGDB|ppa018060m unnamed protein product 69.3 1e-14

gnl|PeGDB|ppa019628m unnamed protein product 68.9 2e-14

gnl|PeGDB|ppa023688m unnamed protein product 68.6 2e-14

gnl|PeGDB|ppa019497m unnamed protein product 66.6 1e-13

gnl|PeGDB|ppa026529m unnamed protein product 65.1 5e-13

gnl|PeGDB|ppa017840m unnamed protein product 65.1 5e-13

gnl|PeGDB|ppa023459m unnamed protein product 64.3 9e-13

gnl|PeGDB|ppa024296m unnamed protein product 63.2 2e-12

gnl|PeGDB|ppa000585m unnamed protein product 62.8 3e-12

gnl|PeGDB|ppa015410m unnamed protein product 62.4 3e-12

gnl|PeGDB|ppa015430m unnamed protein product 62.4 4e-12

gnl|PeGDB|ppa021102m unnamed protein product 62.4 4e-12

gnl|PeGDB|ppa023967m unnamed protein product 62.4 4e-12

gnl|PeGDB|ppa025472m unnamed protein product 61.2 1e-11

gnl|PeGDB|ppa024688m unnamed protein product 60.5 2e-11

gnl|PeGDB|ppb015618m unnamed protein product 58.5 8e-11

gnl|PeGDB|ppa016158m unnamed protein product 58.5 8e-11

gnl|PeGDB|ppa027155m unnamed protein product 57.0 2e-10

gnl|PeGDB|ppa015450m unnamed protein product 57.0 2e-10

gnl|PeGDB|ppa023385m unnamed protein product 55.8 6e-10

gnl|PeGDB|ppa021490m unnamed protein product 55.5 7e-10

gnl|PeGDB|ppa014797m unnamed protein product 53.5 3e-09

gnl|PeGDB|ppa001315m unnamed protein product 52.4 8e-09

gnl|PeGDB|ppa018338m unnamed protein product 52.4 8e-09

gnl|PeGDB|ppa016623m unnamed protein product 50.1 5e-08

gnl|PeGDB|ppa021587m unnamed protein product 49.3 8e-08

gnl|PeGDB|ppa024010m unnamed protein product 48.1 2e-07

gnl|PeGDB|ppa025498m unnamed protein product 47.0 4e-07

gnl|PeGDB|ppa026840m unnamed protein product 47.4 4e-07

gnl|PeGDB|ppa021808m unnamed protein product 47.0 5e-07

gnl|PeGDB|ppa015427m unnamed protein product 45.4 2e-06

gnl|PeGDB|ppa020280m unnamed protein product 45.4 2e-06

gnl|PeGDB|ppa018131m unnamed protein product 45.1 2e-06

gnl|PeGDB|ppb010273m unnamed protein product 43.1 2e-06 PL alone

gnl|PeGDB|ppa026065m unnamed protein product 44.7 2e-06

gnl|PeGDB|ppa020500m unnamed protein product 42.4 5e-06

gnl|PeGDB|ppa024525m unnamed protein product 43.9 5e-06

gnl|PeGDB|ppa016630m unnamed protein product 43.1 1e-05

gnl|PeGDB|ppa023165m unnamed protein product 42.7 1e-05

gnl|PeGDB|ppa026531m unnamed protein product 42.0 2e-05

gnl|PeGDB|ppa022091m unnamed protein product 41.6 3e-05

gnl|PeGDB|ppa022016m unnamed protein product 41.6 3e-05

gnl|PeGDB|ppa016634m unnamed protein product 41.6 3e-05

gnl|PeGDB|ppa021718m unnamed protein product 41.2 4e-05

gnl|PeGDB|ppa015449m unnamed protein product 40.8 5e-05

gnl|PeGDB|ppa014815m unnamed protein product 40.4 7e-05

gnl|PeGDB|ppa000489m unnamed protein product 39.7 1e-04

gnl|PeGDB|ppa021441m unnamed protein product 39.3 2e-04

gnl|PeGDB|ppa023180m unnamed protein product 38.1 3e-04

gnl|PeGDB|ppa000828m unnamed protein product 38.1 4e-04 LRR with PL

gnl|PeGDB|ppa017503m unnamed protein product 36.2 0.001

gnl|PeGDB|ppa021703m unnamed protein product 35.8 0.002

gnl|PeGDB|ppa016162m unnamed protein product 35.8 0.002

gnl|PeGDB|ppa1027179m unnamed protein product 35.8 0.002

gnl|PeGDB|ppa021903m unnamed protein product 35.4 0.003

gnl|PeGDB|ppa025692m unnamed protein product 35.0 0.003

gnl|PeGDB|ppa022940m unnamed protein product 33.9 0.007

gnl|PeGDB|ppa018964m unnamed protein product 33.9 0.008

gnl|PeGDB|ppa1027137m unnamed protein product 32.3 0.027 unknown

gnl|PeGDB|ppa000577m unnamed protein product 31.2 0.064

gnl|PeGDB|ppa019637m unnamed protein product 30.8 0.086 LRR with PL

gnl|PeGDB|ppa021062m unnamed protein product 30.8 0.093

gnl|PeGDB|ppa025310m unnamed protein product 30.4 0.10

gnl|PeGDB|ppa017612m unnamed protein product 30.4 0.11

gnl|PeGDB|ppa019412m unnamed protein product 30.4 0.13

gnl|PeGDB|ppa015956m unnamed protein product 30.0 0.14

gnl|PeGDB|ppa023909m unnamed protein product 29.6 0.21

gnl|PeGDB|ppa024963m unnamed protein product 28.5 0.55

gnl|PeGDB|ppa004644m unnamed protein product 28.1 0.73 GLYCOSYL HYDROLASE

gnl|PeGDB|ppa004387m unnamed protein product 27.7 1.00 GLYCOSYL HYDROLASE

gnl|PeGDB|ppa017041m unnamed protein product 27.7 1.1

gnl|PeGDB|ppa020033m unnamed protein product 27.3 1.6

gnl|PeGDB|ppa004465m unnamed protein product 26.9 1.7 hexose transporter

gnl|PeGDB|ppa023486m unnamed protein product 26.6 2.5

gnl|PeGDB|ppa016853m unnamed protein product 26.6 2.6 hexose transporter

gnl|PeGDB|ppa023229m unnamed protein product 25.8 5.6 hexose transporter

gnl|PeGDB|ppa004219m unnamed protein product 25.0 8.7 SUGAR TRANSPORTER 1

Database: *Sorghum bicolour* peptides

36,338 sequences; 14,066,714 total letters

Query= PL1

Length=95

Score E

Sequences producing significant alignments: (Bits) Value

gnl|SbGDB|Sb03g025600.1 unnamed protein product 28.1 0.78 SUGAR TRANSPORTER 1

gnl|SbGDB|Sb06g029230.1 unnamed protein product 27.3 1.9 disease resistance protein (NBS-LRR class)

gnl|SbGDB|Sb02g033230.1 unnamed protein product 26.2 4.0 aldo/keto reductase

gnl|SbGDB|Sb08g002290.1 unnamed protein product 25.8 7.1 disease resistance protein (NBS-LRR class), putative

gnl|SbGDB|Sb09g021630.1 unnamed protein product 25.4 7.3 hexose transporter

gnl|SbGDB|Sb10g007210.1 unnamed protein product 25.4 8.1 unknown

Database: *Zea mays* peptides

136,770 sequences; 30,594,017 total letters

Query= PL1

Length=95

Score E

Sequences producing significant alignments: (Bits) Value

gnl|ZmGDB|GRMZM2G022440_P01 seq=translation; coord=3:219258608..... 28.9 1.2 transporter activity

gnl|ZmGDB|GRMZM2G159187_P01 seq=translation; coord=2:214936963..... 28.5 1.6 transporter activity

gnl|ZmGDB|GRMZM5G843141_P01 seq=translation; coord=2:43434582..4... 26.9 4.6 transporter activity
