# Supplementary material for: TNL genes in peach: insights into the post-LRR domain
Source: BMC Genomics. 2016 Apr 30;17:317. doi: 10.1186/s12864-016-2635-0 (PMC4851768; doi:10.1186/s12864-016-2635-0)
Supplement: Additional file 4: — Physical map of the TNL-related genes in the peach genome v1.0 and revision observed in v2.0. The map position of the 195 total TNL-related proteins is depicted within the eight scaffolds (chromosomes). Accession numbers are related to the genome glossary of the v1.0. Merged predicted peptides identified through corrected predictions are underlined and highlighted with brackets. The size of each scaffold (in Mb) and the respective number of TNL-related predicted peptides are shown under each scaffold. The Ma orthologue is indicated in red upper case. Unmapped genes in the v1.0 and mapped in the v2.0 are in green boxes. The red box shows re-localisation of TNL genes in the v2.0. Predicted peptides that are not indicated in the v2.0 are labelled with an asterisk. (PDF 167 kb) [file 12864_2016_2635_MOESM4_ESM.pdf]

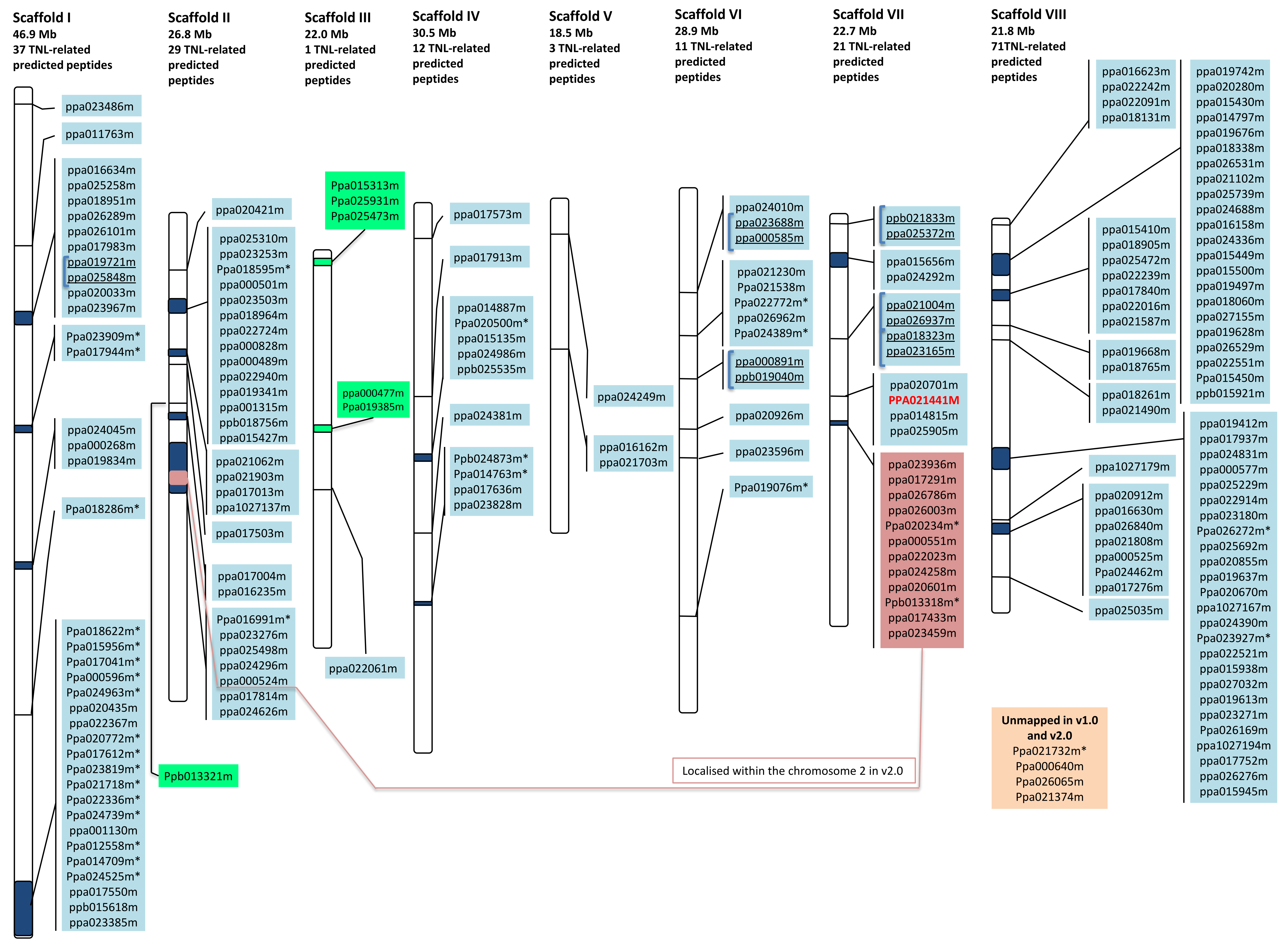

Scaffold I
46.9 Mb
37 TNL-related predicted peptides
ppa023486m
ppa011763m
ppa016634m
ppa025258m
ppa018951m
ppa026289m
ppa026101m
ppa017983m
ppa019721m
ppa025848m
ppa020033m
ppa023967m
Ppa023909m*
Ppa017944m*
ppa024045m
ppa000268m
ppa019834m
Ppa018286m*
Ppa018622m*
Ppa015956m*
Ppa017041m*
Ppa000596m*
Ppa024963m*
ppa020435m
ppa022367m
Ppa020772m*
Ppa017612m*
Ppa023819m*
Ppa021718m*
Ppa022336m*
Ppa024739m*
ppa001130m
Ppa012558m*
Ppa014709m*
Ppa024525m*
ppa017550m
ppb015618m
ppa023385m
Scaffold II
26.8 Mb
29 TNL-related predicted peptides
ppa020421m
ppa025310m
ppa023253m
Ppa018595m*
ppa000501m
ppa023503m
ppa018964m
ppa022724m
ppa000828m
ppa000489m
ppa022940m
ppa019341m
ppa001315m
ppb018756m
ppa015427m
ppa021062m
ppa021903m
ppa017013m
ppa1027137m
ppa017503m
ppa017004m
ppa016235m
Ppa016991m*
ppa023276m
ppa025498m
ppa024296m
ppa000524m
ppa017814m
ppa024626m
Ppb013321m
Scaffold III
22.0 Mb
1 TNL-related predicted peptides
Ppa015313m
Ppa025931m
Ppa025473m
ppa000477m
Ppa019385m
ppa022061m
Scaffold IV
30.5 Mb
12 TNL-related predicted peptides
ppa017573m
ppa017913m
ppa014887m
Ppa020500m*
ppa015135m
ppa024986m
ppb025535m
ppa024381m
Ppb024873m*
Ppa014763m*
ppa017636m
ppa023828m
Scaffold V
18.5 Mb
3 TNL-related predicted peptides
ppa024249m
ppa016162m
ppa021703m
Scaffold VI
28.9 Mb
11 TNL-related predicted peptides
ppa024010m
ppa023688m
ppa000585m
ppa021230m
Ppa021538m
Ppa022772m*
ppa026962m
Ppa024389m*
ppa000891m
ppb019040m
ppa020926m
ppa023596m
Ppa019076m*
Scaffold VII
22.7 Mb
21 TNL-related predicted peptides
ppb021833m
ppa025372m
ppa015656m
ppa024292m
ppa021004m
ppa026937m
ppa018323m
ppa023165m
ppa020701m
PPA021441M
ppa014815m
ppa025905m
ppa023936m
ppa017291m
ppa026786m
ppa026003m
Ppa020234m*
ppa000551m
ppa022023m
ppa024258m
ppa020601m
Ppb013318m*
ppa017433m
ppa023459m
Localised within the chromosome 2 in v2.0
Scaffold VIII
21.8 Mb
71TNL-related predicted peptides
ppa016623m
ppa022242m
ppa022091m
ppa018131m
ppa015410m
ppa018905m
ppa025472m
ppa022239m
ppa017840m
ppa022016m
ppa021587m
ppa019668m
ppa018765m
ppa018261m
ppa021490m
ppa1027179m
ppa020912m
ppa016630m
ppa026840m
ppa021808m
ppa000525m
Ppa024462m
ppa017276m
ppa025035m
ppa019742m
ppa020280m
ppa015430m
ppa014797m
ppa019676m
ppa018338m
ppa026531m
ppa021102m
ppa025739m
ppa024688m
ppa016158m
ppa024336m
ppa015449m
ppa015500m
ppa019497m
ppa018060m
ppa027155m
ppa019628m
ppa026529m
ppa022551m
Ppa015450m
ppb015921m
ppa019412m
ppa017937m
ppa024831m
ppa000577m
ppa025229m
ppa022914m
ppa023180m
Ppa026272m*
ppa025692m
ppa020855m
ppa019637m
Ppa020670m
ppa1027167m
ppa024390m
Ppa023927m*
ppa022521m
ppa015938m
ppa027032m
ppa019613m
ppa023271m
Ppa026169m
ppa1027194m
ppa017752m
ppa026276m
ppa015945m
Unmapped in v1.0 and v2.0
Ppa021732m*
Ppa000640m
Ppa026065m
Ppa021374m
